# Amyloid domains in the cell nucleus controlled by nucleoskeletal protein lamin B1 reveal a new pathway of mercury neurotoxicity

**DOI:** 10.7717/peerj.754

**Published:** 2015-02-05

**Authors:** Florian Arnhold, Karl-Heinz Gührs, Anna von Mikecz

**Affiliations:** 1 IUF—Leibniz Research Institute for Environmental Medicine at Heinrich-Heine-University Duesseldorf, Duesseldorf, Germany; 2 CF Proteomics, FLI—Leibniz-Institute for Age Research, Fritz-Lipman-Institute e.V., Jena, Germany

**Keywords:** Amyloid, Lamin B, Mercury, Ribonucleoproteins, RNA splicing, Protein fibrillation, Proteomics, Nucleus, Nucleoplasm, Confocal microscopy

## Abstract

Mercury (Hg) is a bioaccumulating trace metal that globally circulates the atmosphere and waters in its elemental, inorganic and organic chemical forms. While Hg represents a notorious neurotoxicant, the underlying cellular pathways are insufficiently understood. We identify amyloid protein aggregation in the cell nucleus as a novel pathway of Hg-bio-interactions. By mass spectrometry of purified protein aggregates, a subset of spliceosomal components and nucleoskeletal protein lamin B1 were detected as constituent parts of an Hg-induced nuclear aggregome network. The aggregome network was located by confocal imaging of amyloid-specific antibodies and dyes to amyloid cores within splicing-speckles that additionally recruit components of the ubiquitin-proteasome system. Hg significantly enhances global proteasomal activity in the nucleus, suggesting that formation of amyloid speckles plays a role in maintenance of protein homeostasis. RNAi knock down showed that lamin B1 for its part regulates amyloid speckle formation and thus likewise participates in nuclear protein homeostasis. As the Hg-induced cascade of interactions between the nucleoskeleton and protein homeostasis reduces neuronal signalling, amyloid fibrillation in the cell nucleus is introduced as a feature of Hg-neurotoxicity that opens new avenues of future research. Similar to protein aggregation events in the cytoplasm that are controlled by the cytoskeleton, amyloid fibrillation of nuclear proteins may be driven by the nucleoskeleton.

## Introduction

The heavy metal mercury (Hg) represents a global pollutant that is released predominantly due to anthropogenic activities including artisanal gold mining, metal or cement production, coal-burning and fossil fuel combustion (Streets et al., 2011; UNEP, 2013; Amos et al., 2014). Global circulation of Hg is now inventoried throughout the Pacific and Atlantic oceans (Lamborg et al., 2014) and best reflected by its detection in marine food chains of remote areas such as the Arctic (Bocharova et al., 2013). Seafood-rich diets result in elevated total Hg concentrations of hair, cord blood or human transition milk samples and have been correlated with mild cognitive deficits in cohorts of prenatally exposed school children (Grandjean et al., 1995; Grandjean et al., 1997). While Hg represents an acknowledged neurotoxicant, little is known about the molecular pathways leading to adverse biological end points such as impairment of neural functions and neurodegeneration (Eto, 1997; Axelrad et al., 2007; Korbas et al., 2010). This is particularly noteworthy since inorganic mercury (I-Hg) currently provides the best available marker for assessment of chronic exposures rises in the US population (Laks, 2014).

A prominent molecular pathway of neurodegeneration is amyloid protein fibrillation that occurs (i) spontaneously in systemic amyloidoses and neurodegenerative aggregation diseases (Goedert, 2001; Ross & Poirier, 2004) or (ii) after experimental induction *in vitro*, for example by exposition of biochemically purified proteins or mammalian cell culture to heavy metals (Uversky, Li & Fink, 2001; Chen et al., 2008). Protein aggregates in nuclei of brain cells are a hallmark of a number of neurodegenerative triplet repeat diseases such as Huntington’s disease (HD). In the HD cortex and striatum, intranuclear protein aggregates contain the mutant huntingtin that has a CAG (polyglutamine, polyQ) repeat expansion as well as additional proteins such as ubiquitin, suggesting involvement of proteasomal proteolysis in the formation of amyloid inclusions (DiFiglia et al., 1997). Nuclear co-aggregation of huntingtin, ubiquitin and proteasomes was likewise observed in brains from mice transgenic for the HD mutation (Davies et al., 1997). Notably, nuclei of HD mice display a distinct morphology, since their nuclear envelope changes to an irregular shape (Davies et al., 1997). In addition to components of the ubiquitin-proteasome system, other nuclear proteins such as transcriptional co-activator CREB-binding protein (CBP) and the general transcription factor TATA-binding protein (TBP) were shown to co-aggregate in nuclear protein inclusions of patients with triplet repeat diseases, suggesting that a variety of aggregation-prone proteins contribute to amyloid-like protein fibrillation in the nucleus (Perez et al., 1998; Von Mikecz, 2014).

Intracellular congestion of otherwise soluble proteins to amyloid-like, sodium dodecyl sulfate (SDS)-resistant protein aggregates proceeds in sequential steps. The current conception delineates the transition of globular proteins through conformational changes of native monomers via oligomeric intermediates to highly ordered amyloid fibrils that may be initiated by locally unfolded states of the native protein (Chiti & Dobson, 2009; Knowles, Vendruscolo & Dobson, 2014; Uversky, 2014). Amyloid-like intermediates as well as amyloid fibers are detectable by solid-state nuclear magnetic resonance, electron microscopy and a variety of dyes including Congo red (CR) or Thioflavin T (ThT). Intracellular amyloid can be localized by means of amyloid-specific antibodies (O’Nuallain & Wetzel, 2002) or peptides (Wiesehan et al., 2003) and the aforementioned amyloid-binding dyes. Systems that mimic the process of nuclear protein aggregation/fibrillation *in vitro* by protein over-expression suggest a mechanism of conjoint sequestration of expanded polyQ proteins, unexpanded polyQ proteins and aggregation-prone nuclear proteins (Von Mikecz, 2014).

In this study we identify protein fibrillation to nuclear amyloid as a pathway that is induced by environmentally relevant concentrations of I-Hg. In an unbiased proteomic approach, nuclear amyloid was isolated biochemically and its proteinaceous composition characterized by subsequent mass spectrometry (MS) showing that components of the splicing machinery constitute a major component of an I-Hg-induced nuclear aggregome. In line with this, confocal imaging demonstrates that the core of nuclear speckles, enriched with spliceosomal components, undergoes stepwise protein fibrillation to amyloid microenvironments. I-Hg-induced nuclear amyloid is correlated with altered RNA processing, specific activation of the nuclear ubiquitin-proteasome system and exclusively forms under the control of nucleoskeletal protein lamin B1 that MS analysis likewise identified as a component of the nuclear aggregome. Such interactions between the nucleoskeleton and nuclear protein homeostasis represent a novel molecular pathway of Hg-(neuro)toxicity that reduces neural signalling and clearly resembles the nuclear pathology observed in neurodegenerative aggregation diseases.

## Materials and Methods

### Cell culture

SH-SY5Y cells (American Tissue Culture Collection) were cultured in a humidified atmosphere with 5% CO_2_ at 37 °C. D-MEM/F-12 (1:1) medium, supplemented with 15% FCS and 1% penicillin/streptomycin, was used as growth/proliferation medium. To induce neuronal differentiation, SH-SY5Y cells were cultured in serum-free Neurobasal™ medium with neural cell supplement (B-27), 10 µM retinoic acid, 1% L-glutamine and 1% penicillin/streptomycin (Gibco, Life Technologies) for seven days. All experiments were performed with differentiated, post-mitotic SH-SY5Y cells. HEp-2 cells (American Tissue Culture Collection) were cultured under the same atmospheric conditions in RPMI1640 medium supplemented with 10% fetal calf serum (FCS) and 5% supplement complete (SC). Cells were treated for four hours or as indicated with 25 µM (SH-SY5Y) or 60 µM (HEp-2) I-Hg, e.g., mercuric chloride (HgCl_2_, Merck KGaA). These treatment protocols were established according to results from atomic absorption spectroscopy of nuclear cell fractions (Table 1) and titration of I-Hg concentrations that do not induce cell death (Fig. S1).

**Table 1:** Hg concentrations as determined by atomic absorption spectroscopy (AAS). Total or I-Hg was determined in samples of *in vitro* exposed cells, laboratory animals or humans and wild animals that feed on marine diets. Total or I-Hg was determined in samples of *in vitro* exposed cells (HEp-2), laboratory animals or humans and wild animals that fed on marine diets.

	Sample	*n*	T-Hg (μg/g ww)	T-Hg (μg/g dw)	**Reference**
HEp-2, w/o I-Hg	Cytoplasm	^a^		0.07 ± 0.05	Here
HEp-2, w/o I-Hg	Nucleus	^a^	0.06 ± 0.01	0.14 ± 0.03	Here
HEp-2, I-Hg (60 μM, 4 h)	Cytoplasm	^a^		0.94 ± 0.98	Here
HEp-2, I-Hg (60 μM, 4 h)	Nucleus	^a^	2.74 ± 1.03	6.08 ± 2.29	Here
Monkey *(Macaca fascicularis)*, Me-Hg (50 μg/kg bw, 6 months)	Brain ^b^	4	3.01 ± 0.28 (0.30 ± 0.15) ^c^		(Vahter et al., 1995)
Monkey *(Macaca fascicularis)*, Me-Hg (50 μg/kg bw, 12 months)	Brain ^b^	4	4.32 ± 1.28 (0.43 ± 0.21) ^c^		(Vahter et al., 1995)
Monkey *(Macaca fascicularis)*, Me-Hg (50 μg/kg bw, 18 months)	Brain ^b^	4	4.66 ± 0.57 (1.14 ± 0.61) ^c^		(Vahter et al., 1995)
Human, mother, at parturition, Faroe Islands	Hair	914		4.27 (2.6–7.7) ^d^	(Grandjean et al., 1997)
Human, child, 12 month old, Faroe Islands	Hair	527		1.12 (0.69–1.88) ^d^	(Grandjean et al., 1997)
Human, child, 7 years old, Faroe Islands	Hair	903		2.99 (1.7–6.1) ^d^	(Grandjean et al., 1997)
Arctic foxes (*Vulpes lagopus*), juveniles, Iceland coastal	Hair	6		4.02 ± 1.82	(Bocharova et al., 2013)
Arctic foxes (*Vulpes lagopus*), juveniles, Iceland inland	Hair	5		4.50 ± 1.92	(Bocharova et al., 2013)
Arctic foxes (*Vulpes lagopus*), adult, Iceland coastal	Hair	10		14.52 ± 2.51	(Bocharova et al., 2013)
Arctic foxes (*Vulpes lagopus*), adult, Iceland inland	Hair	6		2.89 ± 1.31	(Bocharova et al., 2013)
Northern Fulmar (*Fulmarus glacialis*), Norway	Liver	15	3.0 ± 2.7		(Knudsen et al., 2007)
Northern Fulmar (*Fulmarus glacialis*), North Pacific	Liver	15		14.2 ± 10	(Kim et al., 1996)
Northern Fulmar (*Fulmarus glacialis*), North Pacific	Kidney	5		6.7 ± 3	(Kim et al., 1996)
Northern Fulmar (*Fulmarus glacialis*), North Pacific	Muscle	5		1.4 ± 1	(Kim et al., 1996)
Northern Fulmar (*Fulmarus glacialis*), North Pacific	Feathers	17		4.8 ± 2.4	(Kim et al., 1996)
Northern fur seal (*Callorhinus ursinus*), Sanricu, Japan	Liver	24		165 ± 132	(Ikemoto et al., 2004)
Northern fur seal (*Callorhinus ursinus*), Sanricu, Japan	Kidney	20		4.4 ± 1.4	(Ikemoto et al., 2004)
Northern fur seal (*Callorhinus ursinus*), Sanricu, Japan	Muscle	20		1.7 ± 0.5	(Ikemoto et al., 2004)
Northern fur seal (*Callorhinus ursinus*), Sanricu, Japan	Hair	20		4.9 ± 1.1	(Ikemoto et al., 2004)

**Notes.**

a 6 ∗ 10^6^ cells/fraction, triplicated experiments.

b T-Hg or I-Hg concentrations in different brain sites (cerebellum, occipital pole, pons, motor strip, frontal pole, thalamus and pituitary) were averaged for this table; obese and control monkeys were excluded.

c I-Hg.

d Interquartile range.

Bw body weight dw dry weight I-Hg inorganic mercury Me-Hg organic mercury T-Hg total mercury ww wet weight

### Cell viability assay

Cells were seeded simultaneously in culture flasks with the same density and left untreated or treated with I-Hg. At the indicated times, cells were trypsinized and counted by hemocytometer (*n* = 100–200). Cell viability was assessed by Trypan blue exclusion.

### Atomic Absorption Spectroscopy (AAS)

HEp-2 cells were cultured as indicated and fractionated according to subcellular compartments as reported previously (Rockel, Stuhlmann & Von Mikecz, 2005). Immunoblot detection of calnexin or SmB/B’ was used to control for purity of cytoplasmic or nuclear fractions, respectively. Equal loading was controlled by Coomassie Brilliant Blue staining. 6 ∗ 10^6^ cells were used in each fraction. Hg concentrations of the cell fractions were determined by cold vapor atomic absorption spectroscopy as described previously (Wilhelm et al., 2003). To calculate the wet weight and the dry weight of the samples, cellular fractions were prepared as described above. The nuclear pellet, e.g., the nuclear fraction resulting from centrifugation and separation of the cytoplasmic supernatant, was defined as wet weight. For determinations of the dry weight, cellular fractions in lysis buffer were heat- and air dried (80 °C) for 6 h.

### Antibodies

The following antibodies were used for immunofluorescence, filter retardation and/or western blot: WO1 amyloid-specific antibody (mouse monoclonal) (O’Nuallain & Wetzel, 2002), beta tubulin (mouse monoclonal, TUB 2.1, Sigma-Aldrich), FUS/TLS (rabbit monoclonal, EPR5812, Abcam), hsc70 (rabbit monoclonal, EP1531Y, Abcam), lamin B (goat polyclonal, C-20, Santa Cruz Biotechnology, Inc.), lamin A/C (mouse monoclonal, Santa Cruz Biotechnology Inc.), nucleolin C23 (mouse monoclonal, MS-3, Santa Cruz Biotechnology Inc.), nucleophosmin B23 (rabbit polyclonal, H-106, Santa Cruz Biotechnology Inc.), U1-70K/SmB/B’ (human serum, ASR53, von Mikecz serum database), ubiquitin (rabbit polyclonal, Sigma-Aldrich), polyQ (mouse monoclonal, 5TF1-1C2 MAB1574, Millipore), pan hnRNP (mouse monoclonal, C-6, Santa Cruz Biotechnology Inc.), calnexin (rabbit polyclonal, Santa Cruz Biotechnology Inc.), 20S proteasome alpha subunits (mouse monoclonal, MCP231, Millipore), 20S proteasome (rabbit polyclonal, kind gift of B Dahlmann), CaMKII alpha (mouse monoclonal, Abcam), CaMKII alpha phospho T286 (rabbit polyclonal, Abcam).

### Imaging

For confocal immunofluorescence (IF), microscopy cells were grown on cover slips to subconfluence, fixed with methanol (−20 °C, 5 min) and permeabilized with ice-cold acetone (−20 °C, 2 min). After washing with PBS, the cells were incubated with the primary antibody for 1 h and washed with PBS. The secondary antibody (conjugated with FITC, Rhodamin or Cy5) was incubated for 45 min. Cells were washed with PBS, covered in mounting medium (Vectashield, Vector Labs) and stored at 4 °C in the dark. To detect intracellular amyloid, methanol/acetone-fixed cells were incubated with amyloid dyes Congo red (Sigma-Aldrich) or Thioflavin T (Sigma-Aldrich) for 10 min. After washing with PBS, cells were covered in mounting medium and stored at 4 °C in the dark. Immunostained or dye-stained cells were imaged using a confocal laser scanning microscope (Fluoview, IX70, Olympus) with a 60x/1.4NA Plan Apo objective. Congo red labelling was recorded with 568 nm excitation and micrographs of single nuclei are presented as pseudocolor intensity map using Metamorph 4.6 software.

### Ca^2+^-imaging

SH-SY5Y cells were differentiated with retinoic acid (RA; Sigma-Aldrich) for five days and for additional seven days with brain-derived neurotrophic factor (BDNF; Sigma-Aldrich). Intracellular Ca^2+^-levels of living cells were visualized by the Fluo-4 Direct™ Calcium Assay Kit (Molecular Probes; Invitrogen) according to the manufacturer’s instructions. Cells were imaged by confocal laser scanning microscopy (Fluoview, IX70, Olympus; 60x/1.4NA Plan Apo objective; low resolution/high speed settings) with 0.5 Hz. Intracellular Fluo-4 intensity was quantified with Metamorph 4.6 software by intensity measurements within regions of interest (ROIs) for each cell and time point. The stimulation response of each cell was calculated by the difference (%) between base-line intensity and maximum peak intensity.

### SDS-PAGE

Cells were cultured, harvested and lysed in loading buffer (0.05 M Trizma-HCl, pH 6.8, 0.1 M DTT, 2% SDS, 0.1% bromophenol blue, 10% glycerin) or fractionated into cytoplasmic and nuclear proteins as reported previously (Rockel, Stuhlmann & Von Mikecz, 2005). Equal numbers of untreated or treated (as indicated) cells were loaded. Proteins were separated by SDS-PAGE (BioRad) and transferred onto nitrocellulose membranes. SDS-PAGE gels were stained with Coomassie brilliant blue overnight and de-stained for 2.5 h. Nitrocellulose membranes were stained with Ponceau S and used for subsequent immunodetection.

### Filter retardation assay

A dot-blot filter retardation assay was applied to isolate SDS-resistant protein aggregates as previously described (Wanker et al., 1999). In brief, cells were washed and harvested in PBS, spun down, and lysed on ice for 30 min in lysis buffer containing 50 mM Tris–HCl, pH 8.8, 100 mM NaCl, 5 mM MgCl_2_, 0.5% Triton X-100, and 1 mM EDTA supplemented with a protease inhibitor cocktail (Sigma-Aldrich). Insoluble material was collected by centrifugation for 5 min at 14,000 rpm in a microfuge (Eppendorf) at 4 °C. Pellets were resolved in DNase buffer containing 20 mM Tris–HCl, pH 8.0, 15 mM MgCl_2_, and 0.5 mg/ml DNase I and incubated for 1 h at 37 °C. Incubation was terminated by adjusting the mixture to 20 mM EDTA, 2% SDS, and 50 mM DTT, followed by heating to 98 °C for 5 min. Protein samples were further diluted in 2% SDS and filtered through a cellulose acetate membrane (pore size 0.2 mm, Whatman) by applying a vacuum on a BIO-DOT device (BioRad), followed by two washing steps with 0.1% SDS.

### Immunodetection

Cellulose acetate filters with trapped SDS-resistant protein aggregates or SDS-PAGE nitrocellulose membranes were blocked in PBS containing 0.5% Tween20 with 5% nonfat dried milk, followed by Western blot detection. Filters were incubated with primary antibodies for one hour, washed with PBS (containing 0.5% Tween20) and incubated with secondary antibodies (conjugated with peroxidase, Dianova) for 45 min. After washing with PBS (containing 0.5% Tween20) detection was performed with Amersham ECL detection reagent according to the manufacturer’s instructions.

### Mass spectrometry—sample preparation

Filter-trapped protein aggregates were eluted from the cellulose acetate membrane and resuspended by overnight incubation in 6 M guanidinium hydrochloride (GuaHCl). Ten µl 100 mM DTT was added to the GuaHCl solution and incubated for one hour at room temperature, followed by addition of 10 µl 500 mM iodoacetamide and an incubation for one hour. Next, samples were dialyzed (Xpress Micro Dialyzer; Scienova) against 25 mM ammonium bicarbonate (AmBic) for at least one hour, followed by a two hour dialysis against digest buffer (8% acetonitrile/25 mM AmBic). The sample solutions were transferred to 0.5 ml Eppendorf tubes and digested with 15 ng trypsin overnight. The samples were dried in a rotary evaporator, dissolved in 20 µl 0.1% formic acid (FA) in 5% acetonitrile, centrifuged for 2 min (14,000 rpm) and the supernatants were transferred to a polypropylene-sample vial. Five µl of each sample were further analyzed (where necessary, 15 µl were used for one measurement).

### Mass spectrometry—measurement (ESI-LC/MS)

For mass spectrometry (MS) analysis the LTQ Orbitrap XL ETD (ThermoScientific) coupled to a nano-HPLC NanoLC 2D System AS 1 (Eksigent) was used. The samples were loaded onto a trap column (Robust Reversed Phase Solid Phase Extraction Trap 100 µm × 40 mm, NanoSeparations) and washed with 30 µl buffer A (5% acetonitrile/0.1% formic acid). The bound analytes were transferred to a separation column (75 µm × 100 mm, NanoSeparations) by applying a linear gradient from 0 to 38% of buffer B (80% acetonitrile/0.1% formic acid) over 76 min. The measured spectra were processed by ProteomeDiscoverer 1.3 (ThermoScientific) and searched against the SwissProt database by Mascot (MatrixScience) with the following search parameters: enzyme—trypsin with two allowed miss cleavages; fixed modification—carbamidomethylation of cysteine; variable modification—oxidation of methionine, phosphorylation of serine and threonine; measurement precision of precursor ions—10 ppm; measurement precision of fragment ions—0.8 Da. Data compilation was performed by ProteomeDiscoverer 1.3 (ThermoScientific) and Scaffold 3 (Proteome Software).

### Aggregome database analysis—subcellular location

The subcellular location of the SDS-insoluble aggregate components identified by MS was defined according to the UniProtKB database. The annotation nucleus was designated as ‘nucleus,’ annotation cytoplasm and annotations as cytoplasmic organelles (i.e., mitochondria) were designated as ‘cytoplasm.’ Proteins with database annotations for both, nucleus and cytoplasm, were subsumed in the category ‘nucleus & cytoplasm.’ For analysis of the human proteome, the complete UniProtKB database was used.

### Aggregome database analysis—protein classification

The PANTHER (Protein ANalysis THrough Evolutionary Relationships) database v8.0 (Mi, Muruganujan & Thomas, 2013) was used to identify the cellular and molecular functions of the SDS-insoluble aggregate components. The annotation PANTHER protein class was used as a basic classification, and if necessary, revised by a manual modification of class names and protein class affiliation, based on literature and UniProtKB references.

### Aggregome database analysis—quantification of protein features

Protein sequence and structure features were extracted from the UniProtKB database. The number of proteins with a feature (%) and the average number/frequency of the feature within proteins were calculated and are presented as fold change of the corresponding values based on the complete human proteome. All calculations were done in Microsoft Excel. Database raw data was imported from the database *.tab file. The corresponding value of each protein feature was identified and listed for each protein. The lists were used to identify the occurrence of a feature in a certain protein and, in a second step, motifs of a respective feature, i.e., the number of features within proteins were quantified. Based on these values, mean values of the different groups (ground state HEp-2, I-Hg-induced HEp-2, ground state SH-SY5Y, I-Hg-induced SH-SY5Y and complete database) are calculated.

### Aggregome database analysis—quantification of protein–protein interaction partners

The number of known interaction partners of each protein was extracted from the HIPPIE (Human Integrated Protein–Protein Interaction rEference) database v1.5 (Schaefer et al., 2012). The database was imported to Microsoft Excel and the overall numbers of known interactions of each protein were calculated. The Mann–Whitney test was used to test for significant differences between the groups using OriginPro 8.6 (Originlab).

### Aggregome database analysis—quantification of splicing components/complexes

SDS-insoluble aggregome components identified by MS analysis were further characterized in a database search of spliceosomal components (Cvitkovic & Jurica, 2013). Proteins were listed according to spliceosomal component families and participation in different spliceosomal complexes/subunits.

### Proteasome activity assays

Proteasomal acitivity in cell fractions was determined by cleavage of the fluorogenic precursor substrate N-Succinyl-Leu-Leu-Val-Tyr-7-amino-4-methylcoumarin (Suc-LLVY-AMC; Affiniti, Exeter, UK). Ten µM substrate was added to cellular fractions (∼1 × 10^6^ cells), and incubated in a reaction buffer (500 mM Hepes, 10 mM EDTA, pH 7.6). Fluorescent increase resulting from degradation of Suc-LLVY-AMC at 37 °C was monitored over time by means of a fluorometer (Fluoroscan Ascent; Thermo Labsystems, Santa Fe, NM) at 340 nm excitation and at 460 nm emission, using free AMC as a standard. Resulting product curves were followed for up to 16 h. Each value of fluorescence intensity represents a mean value obtained from three independent experiments. After 2 h of activity measurement, 10 µM proteasome inhibitor lactacystin (Alexis Biochemicals, San Diego, CA) was applied to the substrate/lysate reaction where indicated.

### RNA interference

Cells were cultured at low density in 6-well plates or on coverslips and transfected with lamin B1 siRNA (30 pmol or 60 pmol, Santa Cruz Biotechnology Inc.) or a control siRNA with scrambled sequence (siRNA scr., 60 pmol, Santa Cruz) according to the manufacturer’s instructions. Twenty-four hours after transfection, siRNA-treated cells were processed for immunoblotting or imaging. Each experiment was performed at least three times.

### Worm cultivation

*C. elegans* wild-type strain N2 was obtained from the Caenorhabditis Genetics Center (CGC, University of Minnesota, USA). Worms were cultured at 20 °C as described before (Brenner, 1974). For I-Hg treatment, synchronized worms at late L4 stage were transferred to liquid S medium containing H_2_O as negative control or 60 µM I-Hg. Worms were left untreated or exposed to I-Hg for 24 h, fixed and stained with Congo red (0.7 mg/ml). Fixation, Congo red staining and imaging was performed as described before (Scharf, Piechulek & Von Mikecz, 2013).

## Results and Discussion

### Environmentally relevant I-Hg concentrations in the nucleus reduces Ca^**2+**^-signalling in neural cells

I-Hg was applied in all experiments as it currently represents the best available marker for chronic Hg-exposure and accumulates within the human body (Laks, 2014). Furthermore, methyl-(Me-)Hg was shown to be de-methylated and persistently deposited in brains of macaques as I-Hg (Table 1) (Vahter et al., 1995). In order to apply environmentally relevant expositions of human cells, differentiated neural SH-SY5Y cells or proliferating HEp-2 cells were titrated with increasing concentrations of I-Hg and the cell viability was analyzed (Fig. S1). Concentrations that did not induce cell death, e.g., 25 µM I-Hg in SH-SY5Y or 60 µM I-Hg in HEp-2 cells, were designated as “mild” and used in subsequent experiments. By atomic absorption spectroscopy (AAS) total Hg was determined in cell fractions that were left untreated or exposed to the higher I-Hg concentration of 60 µM (Fig. S2) and compared with a variety of hair and organ samples from Hg-exposed animals or humans (Table 1). Due to the similar range of total Hg concentrations in nuclear fractions of I-Hg-treated HEp-2 cells, namely 2.74 ± 1.03 µg/g Hg wet weight and 6.08 ± 2.29 µg/g Hg dry weight, as compared with samples from humans and animals that fed on marine diets or experimentally exposed monkeys, we consider the I-Hg concentrations applied in this study environmentally relevant.

**Figure 1:**
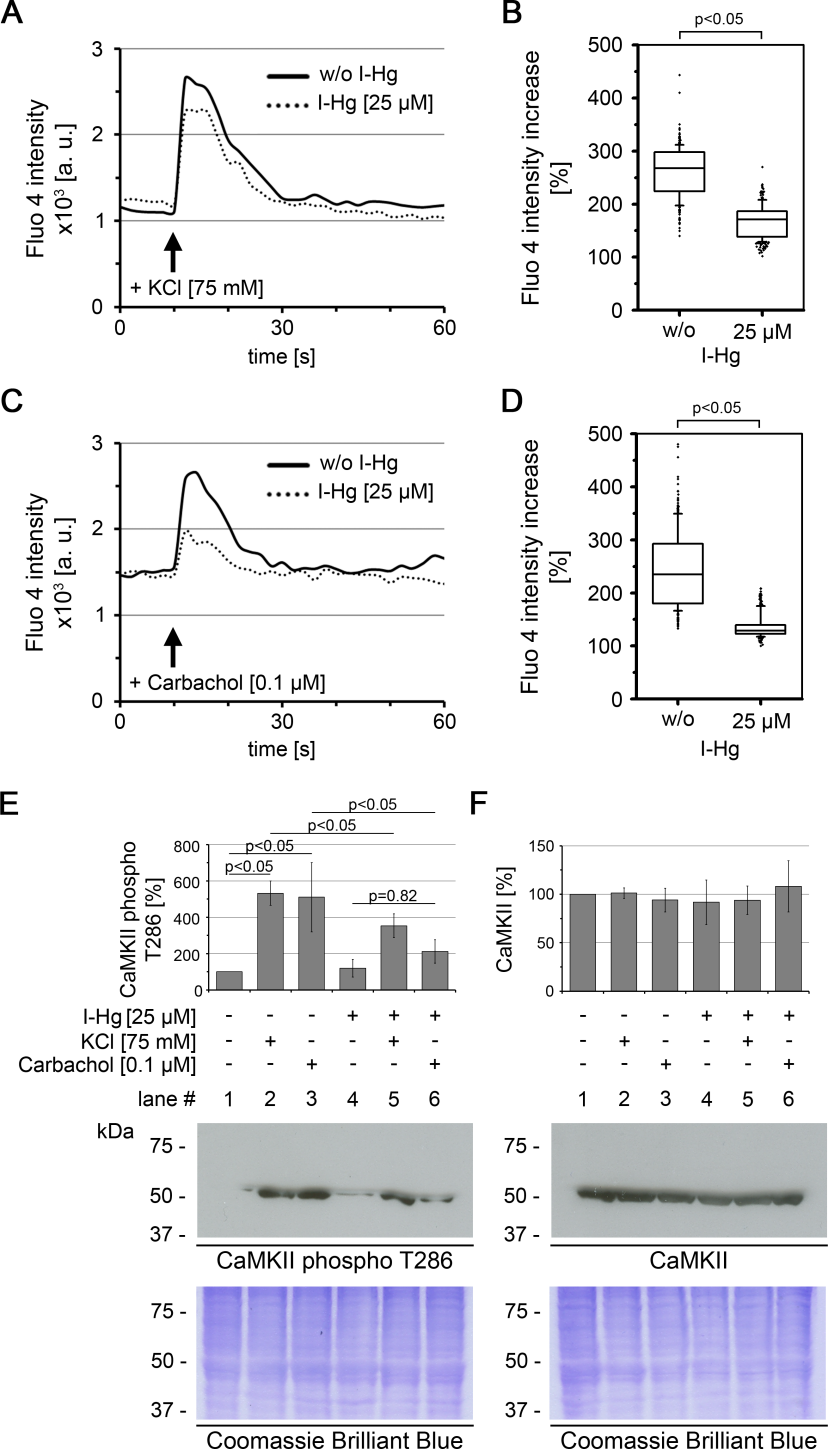
I-Hg reduces Ca^2+^-signalling in SH-SY5Y neurons. Intracellular Ca^2+^ of untreated or I-Hg-treated neural SH-SY5Y cells was monitored by the Fluo-4 DirectTM assay. Cells were imaged by time-lapse confocal laser scanning microscopy with low resolution/high speed settings. After 10 s cells were stimulated with either (A, B) KCl (75 mM) or with (C, D) the cholinergic agonist carbachol (0.1 µM). (A, C) Intracellular fluorescence intensity of representative single cells was measured with MetaMorph software and plotted as intensity (a.u.) over time (s). Continuous curve, untreated SH-SY5Y cells; dashed curve, I-Hg-treated SH-SY5Y cells. (B, D) Ca^2+^-fluctuations were quantified by calculation of the intensity increase after stimulation as percentage (%) of the base-line intensity. Results of three independent experiments were pooled and are presented as box plots (KCl/w/o I-Hg, *n* = 312 cells; KCl/+I-Hg, *n* = 291 cells; carbachol/w/o I-Hg, *n* = 311 cells; carbachol/+I-Hg, *n* = 301 cells). *P*-values of nonparametric Mann–Whitney tests indicate significant differences between untreated and I-Hg-treated cells. (E) Expression of T286-phosphorylated CaMKII and (F) total CaMKII was analyzed by immunoblotting. Untreated or I-Hg-exposed neural SH-SY5Y cells were either left unstimulated or additionally stimulated with KCl or carbachol. Bar graphs show mean values and standard deviations of densitometric analyses of three independent experiments. *P*-values (*p* < 0.05) indicate significant differences (one-way ANOVA with Tukey’s post-hoc test). Corresponding Coomassie Brilliant Blue staining confirms equal protein loading. A.u., arbitrary units; s, seconds.

Usage of differentiated SH-SY5Y cells offers the possibility to investigate effects of mild I-Hg concentrations on the neural system, as the brain represents both (i) one of the main deposition sites of I-Hg and Me-Hg and (ii) a respective target of neurotoxicity in exposed humans and animals (Table 1; (Clarkson & Magos, 2006)). As changes of intracellular Ca^2+^ levels represent an initial event of the neuronal signalling cascade, we next analyzed Ca^2+^-signalling in differentiated SH-SY5Y cells to determine if environmentally relevant concentrations of I-Hg effect neural activity. Ca^2+^-influx was observed after stimulation of SH-SY5Y neurons with KCl or the cholinergic agonist carbachol by measuring the fluorescence intensity of the Ca^2+^-indicator Fluo 4 over time. The addition of both, KCl as well as carbachol, concurred with an immediate peak of Fluo 4 intensity that linearly regressed to the basal level within the next 20 s of the measurements (Figs. 1A and 1C). A difference between untreated (Figs. 1A and 1C; solid line) and I-Hg-treated (dotted line) SH-SY5Y cells was identified as I-Hg-exposure reduced the peak intensity under conditions of KCl or carbachol stimulation significantly (Figs. 1B and 1D). Notably, I-Hg reduces the carbachol stimulated Ca^2+^-influx to a higher extent than the response to stimulation by KCl, suggesting that neuro-specific signalling is more susceptible.

To characterize the downstream signalling cascade of neural processing, we analyzed the activation of the protein Ca^2+^/calmodulin-dependent protein kinase II (CaMKII) (Figs. 1E and 1F). CaMKII regulates long-term potentiation and is activated by phosphorylation of the tyrosine residue at position 286 (T286) (Malinow, Madison & Tsien, 1988). Immunoblotting shows that after stimulation of untreated SH-SY5Y neurons with KCl or carbachol expression of T286-phospho CaMKII is significantly induced by about 5-fold (Fig. 1E, lanes 1–3). In contrast, a significantly reduced activation of CaMKII phosphorylation was observed in I-Hg-treated SH-SY5Y neurons (Fig. 1E, lanes 4–6). Consistent with the results from intracellular Ca^2+^-imaging carbachol stimulation is more susceptible to I-Hg-exposure, since expression of T286-phospho CaMKII is not significantly induced in comparison with unstimulated neurons, e.g., I-Hg impairs activation of CaMKII by phosphorylation (Fig. 1E, lanes 4 and 6). Stimulation-specific activation of phosphorylated CaMKII was corroborated by comparison with total CaMKII that showed equal expression in all conditions of I-Hg-treatment with or without modulation of Ca^2+^-influx by KCl or carbachol (Fig. 1E, lanes 1–6). Taken together, we show that environmentally relevant concentrations of I-Hg interfere with initial and downstream steps of the Ca^2+^-signalling cascade, thereby exerting adverse effects on neural function by reduction of the excitation plasticity. Further studies are needed to investigate in detail the nature of the interactions between I-Hg accumulation in the cell nucleus (Table 1) and failure of neuronal signaling, e.g., supposable direct interactions of I-Hg with neuronal receptors and synaptic function.

### Amyloid microenvironments are induced by environmentally relevant concentrations of I-Hg

**Figure 2:**
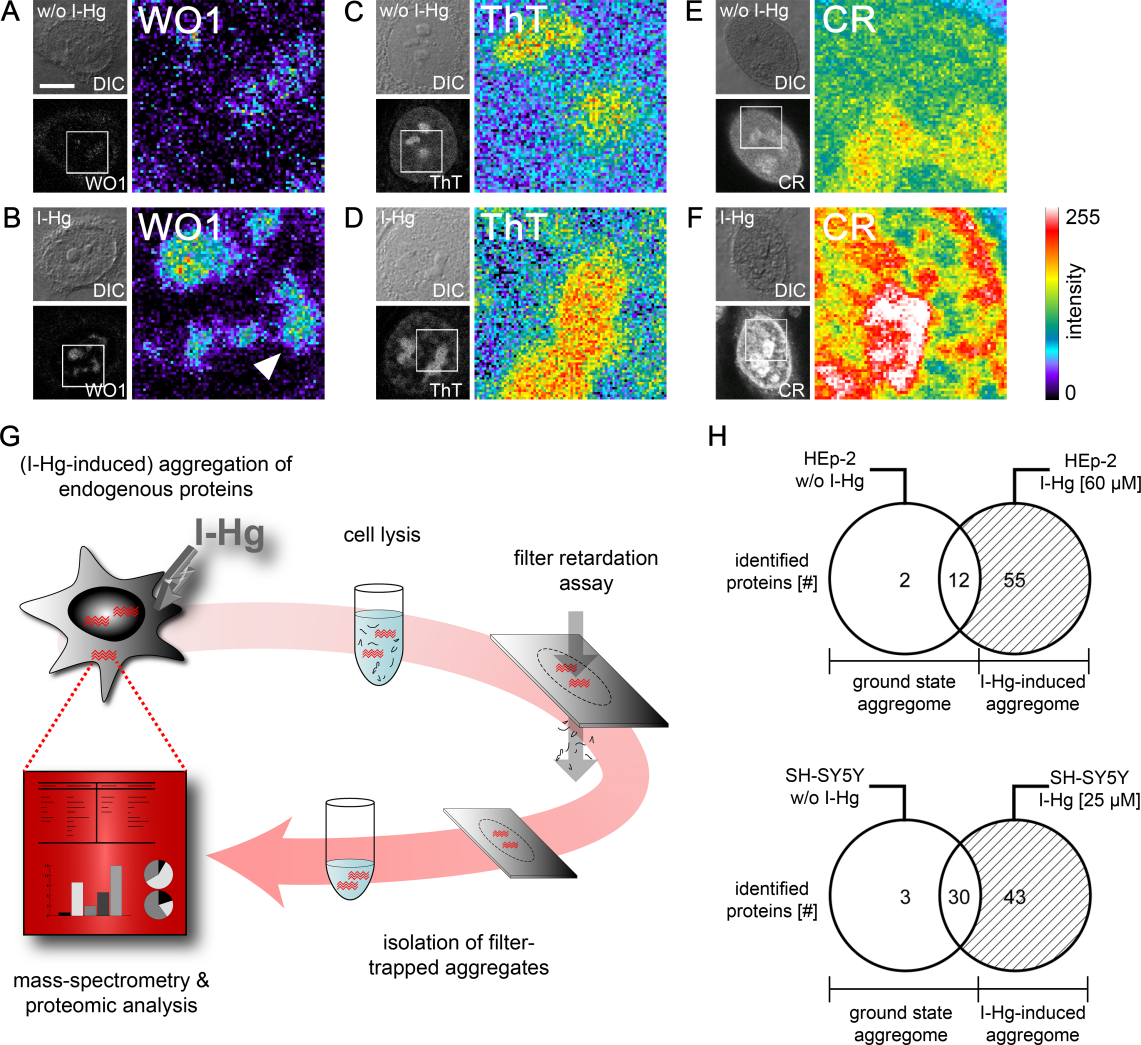
Mild concentrations of inorganic mercury increase local and global amyloid fibrillation in the cell. Untreated or 4 h I-Hg-treated HEp-2 cells were fixed and stained for intracellular amyloid. (A–F) Micrographs show confocal imaging of representative cells in differential interference contrast (DIC) or fluorescence channels. Blow ups show respective regions of the cell nucleus of the fluorescence channel visualized with pseudocolor (Metamorph) that indicates intensity of fluorescence (blue/green: low intensity, red/white: high intensity). Representative cells were immunolabelled with the amyloid-specific antibody WO1 and FITC-conjugated secondary antibodies w/o (A) or with I-Hg-exposure (B). Cells were stained with the amyloid dyes ThT (C, D) or CR (E, F). Note the pattern change of the dyes in the nucleoplasm after induction of protein fibrillation by I-Hg (D, F). (G) Isolation and characterization of endogenous protein aggregates (schematic): human cells, i.e., proliferating HEp-2 or post-mitotic retinoic acid (RA)-differentiated, neural SH-SY5Y, were left untreated to investigate the ground state of protein aggregation or treated for 4 h with I-Hg to induce an advanced step of endogenous protein fibrillation. Cells were lysed and SDS-insoluble protein aggregates isolated by filter retardation assays. Aggregates were eluted from the filters, re-suspended in 6M guanidinium-HCl and resulting samples analyzed by mass spectrometry (ESI-LC/MS). MS results were interpreted by means of proteomic, database-supported quantification. (H) The numbers of SDS-insoluble aggregate components in untreated and 4 hour I-Hg-treated HEp-2 cells or SH-SY5Y neurons are presented as Venn diagrams. Open sets represent the ground state of protein fibrillation, whereas hatched sets represent the induced fibrillation state. Respective intersections show aggregate components that coexist in ground and induced state aggregomes. Complete lists of candidates are available in Tables S1 and S2. CR, Congo red; FITC, fluorescein isothiocyanate; h, hours; I-Hg, inorganic mercury; RA, retinoic acid; ThT, Thioflavin T; w/o, without. Bar, 5 µm.

Due to previous reports that metals induce protein fibrillation *in vitro* (Uversky, Li & Fink, 2001), local amyloid was imaged after I-Hg exposition in human epithelial HEp-2 and neural SH-SY5Y cells by confocal microscopy using different detection reagents. The monoclonal antibody WO1 recognizes conformational epitopes of all amyloid fibrils and amyloid-like aggregates (O’Nuallain & Wetzel, 2002). In the nucleus, WO1 labels nucleoli and speckled domains in the nucleoplasm (Figs. 2A and 2B). The staining pattern is of low intensity in untreated or ground state HEp-2 cells (Fig. 2A), but distinct and of high intensity in cells exposed to I-Hg (Fig. 2B). Notably, environmentally relevant concentrations of I-Hg induce the aggregation of soluble proteins or oligomers in amyloid-like nuclear microenvironments, while cell viability remains unchanged (Fig. S1). In agreement with these results, staining of I-Hg-treated cells with amyloid-binding dyes Thioflavin T (ThT) and Congo red (CR) showed intensified labelling of nucleoli and the nucleoplasm (Figs. 2D and 2F). In contrast to immunolabelling with WO1, the dyes ThT and CR undergo a pattern change in the nucleoplasm from a diffuse homogeneous to an intensified, speckled staining pattern that might indicate the transition of the protein fibrillation state from oligomeric intermediates throughout the nucleoplasm to formation of amyloid-like protein aggregates in distinct nuclear microdomains. Consistent with this idea, WO1 that detects highly ordered amyloid fibrils but not oligomeric intermediates (O’Nuallain & Wetzel, 2002) exclusively labels amyloid microenvironments with a speckled phenotype in the nucleoplasm (Fig. 2B; arrowhead). In agreement, we previously showed by means of filter retardation assays that WO1 selectively detects different protein fibrillation states in the nucleus (Arnhold & von Mikecz, 2011).

The property of I-Hg to induce intracellular protein aggregation was confirmed *in vivo*, e.g., in adult hermaphrodite *C. elegans* nematodes. Worms were left untreated or exposed to 60 µM I-Hg for 24 h (Fig. S3) and stained for amyloid-formation by means of the amyloid-specific dye Congo red. While untreated worms show a weak staining, a discontinuous, high-intensity pattern indicating protein fibrillation is observed in I-Hg-exposed *C. elegans* (Fig. S3). These Congo red binding domains are localized in the cell nucleus, especially in nucleoli and at the nuclear rim.

Heavy metal-induced protein fibrillation has been previously reported for (i) lead and A*β* (Basha et al., 2005), (ii) aluminum, iron, cobalt or copper and *α*-synuclein (Uversky, Li & Fink, 2001) and (iii) mercury and Tau (Yang et al., 2010). Our results indicate that endogenous nuclear proteins likewise have the propensity to aggregate into higher order amyloid-like conformations. Measurement of intracellular (total) Hg by means of atomic absorption spectroscopy (AAS) shows that the heavy metal concentrates in the nucleus (Table 1), which enables direct interactions between Hg-ions and nuclear proteins. The detection of nuclear amyloid in a physiological ground state as well as under conditions of mild I-Hg-exposition raises the question about functional versus adverse, i.e., pathogenic, roles of nuclear protein fibrillation. Here, identification of the nuclear proteins that undergo protein fibrillation and become components of amyloid-like microenvironments may lead to a better understanding.

### Protein composition of I-Hg-induced aggregates

We next asked how to identify components of amyloid-like nuclear protein aggregates and chose a filter assay developed by Scherzinger et al. (1997). The filter retardation assay traps SDS-insoluble protein aggregates on cellulose acetate filters, whereas soluble proteins pass through the 0.2 µm sized pores. In order to distinguish single components of nuclear protein aggregates, we eluted the retained protein fraction from the filters and characterized the proteins in an unbiased approach by mass spectrometry (Fig. 2G). Mass spectrometry of SDS-insoluble protein aggregates was performed as detailed in the Methods section. HEp-2 cells that were left untreated have a ground state aggregome that contains 14 proteins with mainly cytoskeletal or chromatin-organizing functions (Fig. 2H; Table S1). After four hours of exposition to I-Hg, the number of SDS-resistant aggregate components increases to a total of 69 (Fig. 2H). Among these, 55 proteins represent specifically induced aggregate components that are mainly nuclear and involved in organization of chromatin and gene expression (Table S1). In order to validate the results in neural cells, the analysis of SDS-resistant aggregate components by mass spectrometry was replicated in differentiated, post-mitotic SH-SY5Y cells (Fig. 2H; Table S2). In contrast to HEp-2 cells, the ground state aggregome of SH-SY5Y neurons already consists of 33 proteins that are predominately nuclear and participate in chromatin organization or RNA processing. Induction of protein fibrillation by I-Hg results in an aggregome consisting of 76 proteins in total, from which 43 proteins are specifically recruited to the SDS-resistant protein fraction (Fig. 2H; Table S2).

To validate the MS analysis, filter retardation assays and subsequent immunoblotting were performed with representative candidates (Fig. S4). All candidates that specifically occur in the induced aggregome likewise showed an increased signal, i.e., were trapped on cellulose acetate filters after induction of protein fibrillation in neural SH-SY5Y by I-Hg. In contrast, beta tubulin is present in both the ground state and the induced aggregome and is retained in the filter assays of ground state as well as I-Hg-treated SH-SY5Y, respectively (Fig. S4A, top). To monitor protein aggregation, the mouse monoclonal antibody 1C2 against a linear and extended conformation of polyQ repeats (Klein et al., 2013) was used showing that endogenous CAG-repeat proteins are recruited to I-Hg-induced protein aggregates (Fig. S4A, bottom). In order to exclude increased gene expression of representative candidates as a cause for their increased retardation on the filters respective immunoblot analyses of the total cell fractions were carried out. Figure S4B shows that representative aggregome components are equally expressed in total cell fractions of both fibrillation states, namely ground state and I-Hg-induced SH-SY5Y neurons corroborating the specificity of the filter trap results.

Additional confirmation of single candidates comes from aggregomes that were previously characterized using (over)expression of ectopic amyloid proteins to analyze their interactions with the endogenous proteome. A study that reports induction of protein aggregation by expression of ectopic, EGFP-fused, CAG-expanded (Q150) huntingtin exon1 in murine Neuro2A cells shows that the RNA-binding protein FUS is a major component of nuclear polyQ aggregates (Doi et al., 2008). Notably, FUS is likewise a component of I-Hg-induced nuclear protein aggregates in both cell types HEp-2 and SH-SY5Y. Lamin B1 and the AAA^+^ ATPase ruvB-like 1 that both are recruited to the I-Hg-induced aggregome were previously identified as interacting with artificial *β* sheet proteins (Olzscha et al., 2011), corroborating the participation of these candidates in processes of intracellular protein fibrillation.

In line with this is the biological replication, i.e., comparison of the ground state and I-Hg-induced aggregome in proliferating HEp-2 with neural SH-SY5Y cells. Respective results are summarized in a Venn diagram showing that the total number of exclusive ground state and I-Hg-induced aggregate components is similar in HEp-2 (*n* = 69) and SH-SY5Y (*n* = 76) cells (Fig. 2H). However, both cell lines differ concerning the intersection between ground state and I-Hg-induced state. In SH-SY5Y neurons the number of aggregate components that overlap between the ground state and I-Hg-induced aggregome is nearly threefold compared with HEp-2, i.e., 30 versus 12 proteins (Fig. 2H). Thus, studies of single aggregome components are required to identify patterns of protein fibrillation that indicate respective pathways.

Consistent with this idea, ubiquitin occurs as a unique component of the endogenous aggregome in I-Hg-induced SH-SY5Y neurons (Table S2). The fact that ubiquitin likewise constitutes a diagnostic component of neural nuclear inclusions in brains of patients with triplet (CAG) repeat diseases (Ross & Poirier, 2004) corroborates I-Hg-induced SH-SY5Y neurons as a valid model for the characterization of protein fibrillation pathways in the cell nucleus and suggests involvement of the ubiquitin-proteasome system. The presence of heat shock proteins in the I-Hg-induced HEp-2 and SH-SY5Y aggregomes and ubiquitin in the I-Hg-induced SH-SY5Y aggregome (Tables S1 and S2) as well as previous results demonstrating degradation of spliceosomal components U1-70k, SmB/B’ and splicing factor SC35 by the ubiquitin-proteasome system (Rockel, Stuhlmann & Von Mikecz, 2005) prompted us to investigate if amyloid-like microenvironments in the nucleoplasm contain components of the ubiquitin-proteasome system or respective proteolytic activity. Confocal immunofluorescence double labelling shows colocalization of WO1-positive amyloid speckles with 20S proteasomes (Fig. S5B, yellow, filled arrowhead). The respective line scans indicate nearly perfect colocalization (Fig. S5B open arrowhead) suggesting that amyloid speckles recruit proteasomes and may represent proteolytic centers. Consistent with this idea analysis of global proteasomal activity indicated that I-Hg-induced protein fibrillation specifically activates the nuclear ubiquitin-proteasome system (*p* < 0.05) (Fig. S6B), probably to counteract further protein aggregation and maintain protein homeostasis in the nucleus. Formation of amyloid-like nuclear inclusions that contain proteolytic activity was shown previously after exposition of cells with another xenobiotic, namely silica nanoparticles (Chen et al., 2008). A significant subpopulation (30%) of silica-nanoparticle-induced amyloid-like inclusions were identified as proteolytically active supporting the idea that amyloid-domains in the nucleus represent sites of proteasomal protein degradation.

### Overrepresentation of cross-links and protein interactions in components of nuclear protein aggregates

**Figure 3:**
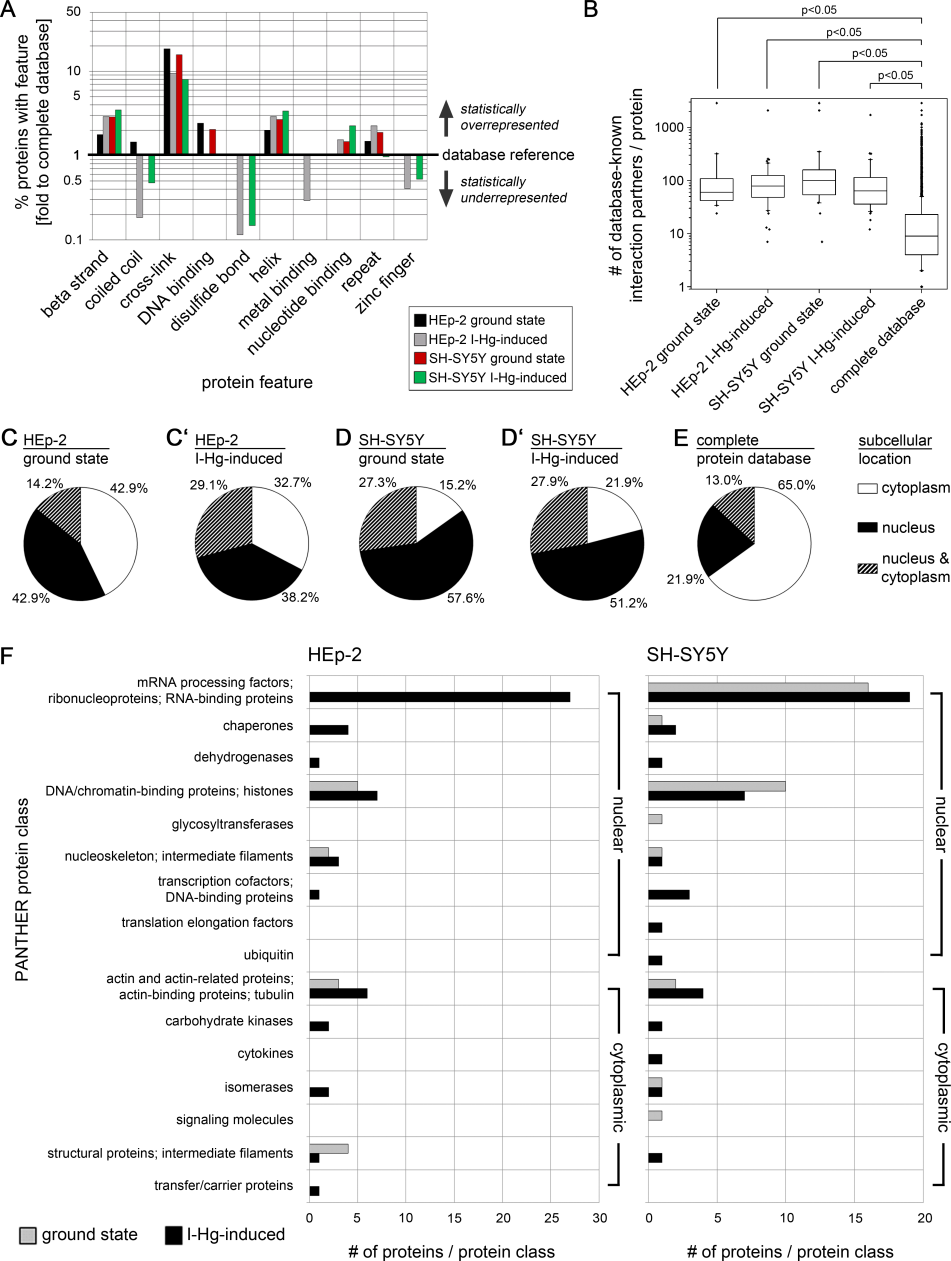
Aggregome components have a propensity for protein interactions and are mainly nuclear. Aggregated proteins show a statistical overrepresentation of cross-links (A) and a higher number of potential interaction partners (B) as compared to the complete proteome database. Sequence features were extracted from the UniProtKB database. Identified components of HEp-2 ground state (black), HEp-2 I-Hg-induced (grey), SH-SY5Y ground state (red) and SH-SY5Y I-Hg-induced (green) aggregates were analyzed. (A) The number of proteins with particular features (%) is calculated and presented as fold change to corresponding values of the complete human proteome database. The database reference line (*y*-value = 1) represents the value of a feature in the complete UniProtKB protein database. Corresponding values above 1 (upward bars) indicate statistical overrepresentation and values below 1 (downward bars) indicate underrepresentation. Values of features that are not found in a single sample (value = 0) are not depicted. (B) The HIPPIE database was used to calculate the number of potential interaction partners of aggregate components listed in Tables S1 and S2. Pooled data is presented as box plots. Nonparametric Mann–Whitney test analyzes significant differences between different groups. *p*-values below 0.05 indicate significance. (C–E) The cell nucleus is a major target of protein aggregation. Parts (C–D’) show the subcellular location of proteins identified by mass spectrometry according to UniProtKB database entries. Data is calculated as percentage of each group and presented as pie charts for aggregate components of (C) HEp-2, ground state, (C’) HEp-2, I-Hg-induced protein fibrillation state, (D) SH-SY5Y, ground state and (D’) SH-SY5Y, I-Hg-induced protein fibrillation state, in comparison with (E) the complete UniProtKB protein database as reference. (F) shows classification of the identified proteins by application of the PANTHER database. The absolute numbers of proteins in each PANTHER database protein class are depicted as bar graphs of HEp-2 cells or SH-SY5Y neurons (two pooled experiments per group; compare with Tables S1 and S2). Analysis of aggregate components is subdivided into ground state (grey bars) or I-Hg-induced protein fibrillation (black bars). Where necessary, the original PANTHER class identifiers were modified and adjusted to the experimental data by manual customization.

We searched next for unifying protein sequence features of the aggregome components that were identified by mass spectrometry. For this purpose, the presence of selected features in aggregated proteins were quantified and compared to the mean value of all database-saved proteins (database reference). This analysis shows a two- to three-fold higher presence of beta strands and helix structures in aggregated proteins compared with statistical expectations from the average protein population (Fig. 3A). Cross-links especially stand out, with an up to 20-fold overrepresentation. Up to two-fold overrepresentation was observed for DNA- and nucleotide binding as well as for sequence repeats. In contrast, zinc finger structures and disulfide(SH)-bonds are underrepresented, which refutes the assumption that treatment with I-Hg induces random protein aggregation by its affinity to SH-groups and argues for isopeptide bonds as likely constituents of the overrepresented cross-links. Surprisingly, coiled coil structures, which are intrinsically aggregation-prone (Fiumara et al., 2010), are underrepresented in aggregome components. In summary, SDS-insoluble aggregome components mainly differ from the global proteome concerning cross links that are even more enriched in the ground state of protein fibrillation (Fig. 3A). In order to investigate whether only the presence or the quantity of a feature within a protein is characteristic for aggregome components, the number of respective features within one protein was extracted from the database and compared with the global proteome (Fig. S7A). The results suggest that the mere presence of a specific protein structure or sequence feature is characteristic for aggregated proteins irrespective of the quantity of the feature within each single protein.

The analysis of protein features indicates a statistically elevated chance that aggregated proteins have cross-links, *β* strands and helices as well as nucleotide-binding capability in comparison with other cellular proteins. These features are important for protein–protein interactions and formation of multi-protein complexes. Therefore, we investigated the interaction propensity of the candidates identified by mass spectrometry. To this end database-listed interaction partners of each aggregate component (compare Tables S1 and S2) were quantified, pooled and presented as box plots. All aggregate components show a median value of 60–100 known interaction partners per protein with a few extreme values that reach up to 3,000 partners (Fig. 3B). Thus, aggregate components interact more frequently in comparison with the whole protein population listed in the database that has a median value of only eight interaction partners per protein. The non-parametric Mann–Whitney test shows that proteins of the I-Hg-induced aggregome have a significantly higher number of interaction partners (Fig. 3B; *p* < 0.05). This supports the idea that aggregate components are proteins with elevated structural interaction capabilities and propensity for complex formation, have a higher number of interaction partners and are therefore prone to form insoluble aggregates. It is tempting to speculate that a certain threshold of protein aggregation might already occur in the ground state of the cell as a functional feature of intracellular crowding and protein homeostasis; however, this affect more and different proteins under cellular stress, i.e., if proteostasis is altered by I-Hg (Figs. S5 and S6). Highly structured aggregate components that have many protein interaction partners are likely constituents of multi-protein complexes and contributors to overcrowded microenvironments and protein fibrillation.

### Overrepresentation of nuclear proteins in protein aggregates

Next, the subcellular localization of I-Hg-induced aggregome components was analyzed. Proteins were defined according to the database UniProtKB in three categories as located in the nucleus or in the cytoplasm or localized in both nucleus and cytoplasm. In ground state HEp-2 42.9% nuclear and 42.9% cytoplasmic aggregome components distribute equally within the two compartments, whereas a subpopulation of 14.2% proteins are both nuclear and cytoplasmic (Fig. 3C). After induction of protein fibrillation by I-Hg, the percentage of nuclear proteins and cytoplasmic proteins is decreased to 38.2% and 32.7%, respectively, while aggregome components that occur in the nucleus as well as in the cytoplasm increase to 29.1% (Fig. 3C’). Neural SH-SY5Y cells have a clear predominance of nuclear aggregome components in both fibrillation states, i.e., 57.6% in ground state and 51.2% in the I-Hg-induced state (Figs. 3D and 3D’). The overrepresentation of nuclear proteins in all examined aggregomes clearly shows when protein location is compared with the complete UniProtKB database where only 21.9% of the proteins are nuclear, 65.0% are cytoplasmic and 13.0% are defined as occurring in both cellular compartments (Fig. 3E). These results indicate that the nucleus is a major target for protein aggregation. Nuclear characteristics such as protein crowding (Munishkina et al., 2008; Hancock, 2004) and organization into domains of concentrated chromatin, molecular machines and ribonucleoprotein complexes (Rouquette et al., 2010; Hemmerich, Schmiedeberg & Diekmann, 2011) may contribute to the facilitated formation of SDS-resistant protein aggregates and amyloidogenic microenvironments (Figs. 2A–2F), respectively.

### Spliceosomal components are a major constituent of I-Hg-induced protein aggregates

A PANTHER databank analysis was performed in order to group proteins of the I-Hg-induced aggregome according to their molecular and cellular function. In Fig. 3F, aggregome components are assigned to molecular and cellular functions, i.e., PANTHER classes, showing that in ground state HEp-2 cells protein aggregation is restricted to chromatin-binding proteins, mainly histones, intermediate filaments and other cytoskeletal proteins (Fig. 3F, first column, grey bars). After induction of an advanced protein fibrillation state by I-Hg, the aggregome additionally contains proteins involved in RNA processing, chaperones, nucleoskeleton, a transcriptional cofactor and a variety of enzymes (Fig. 3F, first column, black bars). With 27 proteins, the RNA processing factors constitute the largest functional group of the I-Hg-induced aggregome.

A similar distribution of functional classes is observable in neural SH-SY5Y cells. The prevalence of RNA processing factors and chaperones occurs in both fibrillation steps, e.g., ground and I-Hg-induced state (Fig. 3F, second column, gray or black bars, respectively). In contrast to HEp-2 cells transcriptional coactivators are a functional class that is specifically induced in SH-SY5Y neurons. With 16 proteins in ground state and 19 proteins in the I-Hg-induced fibrillation state RNA processing factors again stand out as the largest functional class corroborating the results obtained from I-Hg-induced HEp-2 cells. This functional class contains spliceosomal components such as heterogeneous ribonucleoprotein particles (hnRNPs), serine/arginine rich (SR) proteins, splicing factors and RNA helicases that participate in a variety of splicing complexes and processes. Thus, processing and maturation of RNA is identified as a major target of I-Hg-induced protein aggregation in the nucleus which raises the question of interactions between nuclear protein fibrillation and splicing. Notably, the nucleoskeletal protein lamin B1 is a constituent of both I-Hg-induced aggregomes, e.g., in HEp-2 and SH-SY5Y cells.

### Data bank analysis of spliceosomal aggregate components

Next, we used the spliceosome database (Cvitkovic & Jurica, 2013) for detailed characterization of the I-Hg-aggregome candidates that belong to the functional class of RNA processing factors. Table 2 lists aggregome components from HEp-2 cells and SH-SY5Y neurons in ground state or I-Hg-induced fibrillation states with respect to their molecular weight, class/family and participation in different spliceosomal complexes. Splicing occurs as a cascade of subsequent steps that are performed by spliceosomal E, A, B, B* and C complexes (Wahl, Will & Lührmann, 2009). These complexes contain small nuclear ribonucleoproteins (snRNP) and non-snRNP proteins that assemble stepwise on pre-mRNA and undergo major structural rearrangements during the process. Previous MS analyses of spliceosomal complexes indicated that single spliceosomal assembly intermediates consist of 150–300 proteins (Zhou et al., 2002).

**Table 2:** RNA processing factors that are components of the I-Hg-induced aggregome according to their occurence in different RNA maturation/splicing steps. Aggregate components were listed by means of a spliceosome database (Cvitkovic & Jurica, 2013) according to their role in spliceosomal complexes. Positive identifications are marked by a black dot.

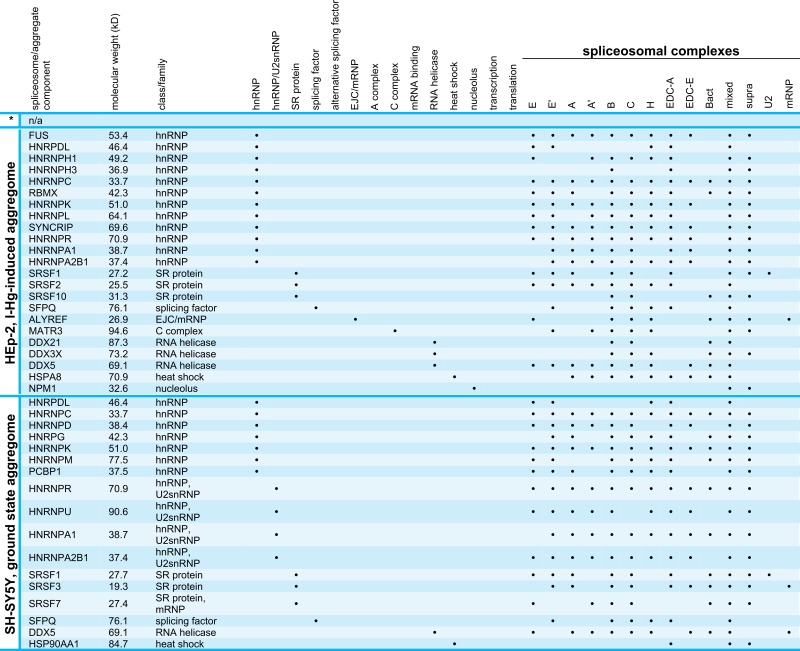
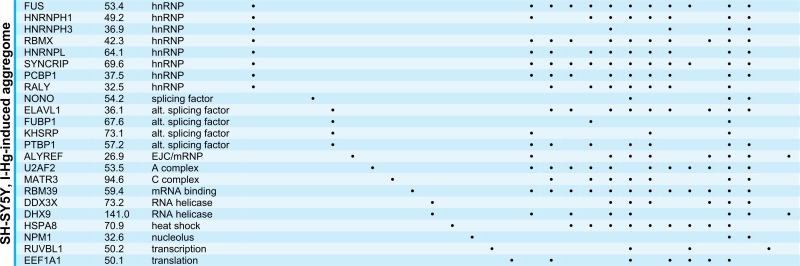

**Notes.**

* HEp-2 ground state aggregome.

Our database analysis of the aggregome components belonging to the PANTHER class of RNA processing shows that they are represented in all families/classes of spliceosome-associated proteins and cover all steps of the splicing cascade (Table 2). In ground state HEp-2 cells we did not detect any SDS-insoluble aggregated spliceosomal components, whereas the I-Hg-induced HEp-2 aggregome recruits spliceosomal components from all families/classes except hnRNP/U2snRNP, alternative splicing factors, and mRNA binding proteins (Table 2). While the neural SH-SY5Y ground state aggregome harbors a repertoire of splicing-associated proteins that participate in a reduced subset of families/classes, the I-Hg-induced fibrillation state recruits spliceosomal components from all families except hnRNP/U2snRNP and SR proteins. This represents a notable switch between ground state and I-Hg-induced protein fibrillation in SH-SY5Y, since in the ground state hnRNP/U2snRNP and SR proteins participate in the SDS-insoluble aggregome (Table 2).

Consistent with this, new questions emerge. What does I-Hg-induced aggregation of certain subsets of spliceosomal components imply for the delicate and complex splicing network? Are single spliceosomal components with key roles in the formation of RNA/RNP structures and/or catalysts of splicing segregated in I-Hg-induced nuclear amyloid? Key components of RNA processing such as the RNA helicases are essential for spliceosomal RNA-RNA rearrangements or RNP remodeling events (Staley & Guthrie, 1998) and thus amyloid fibrillation of these proteins might induce altered splicing. Consistently, the aggregomes identified in this study may represent snap shots of ongoing splicing processes, i.e., their modification.

### Spliceosomal components colocalize with nuclear amyloid

**Figure 4:**
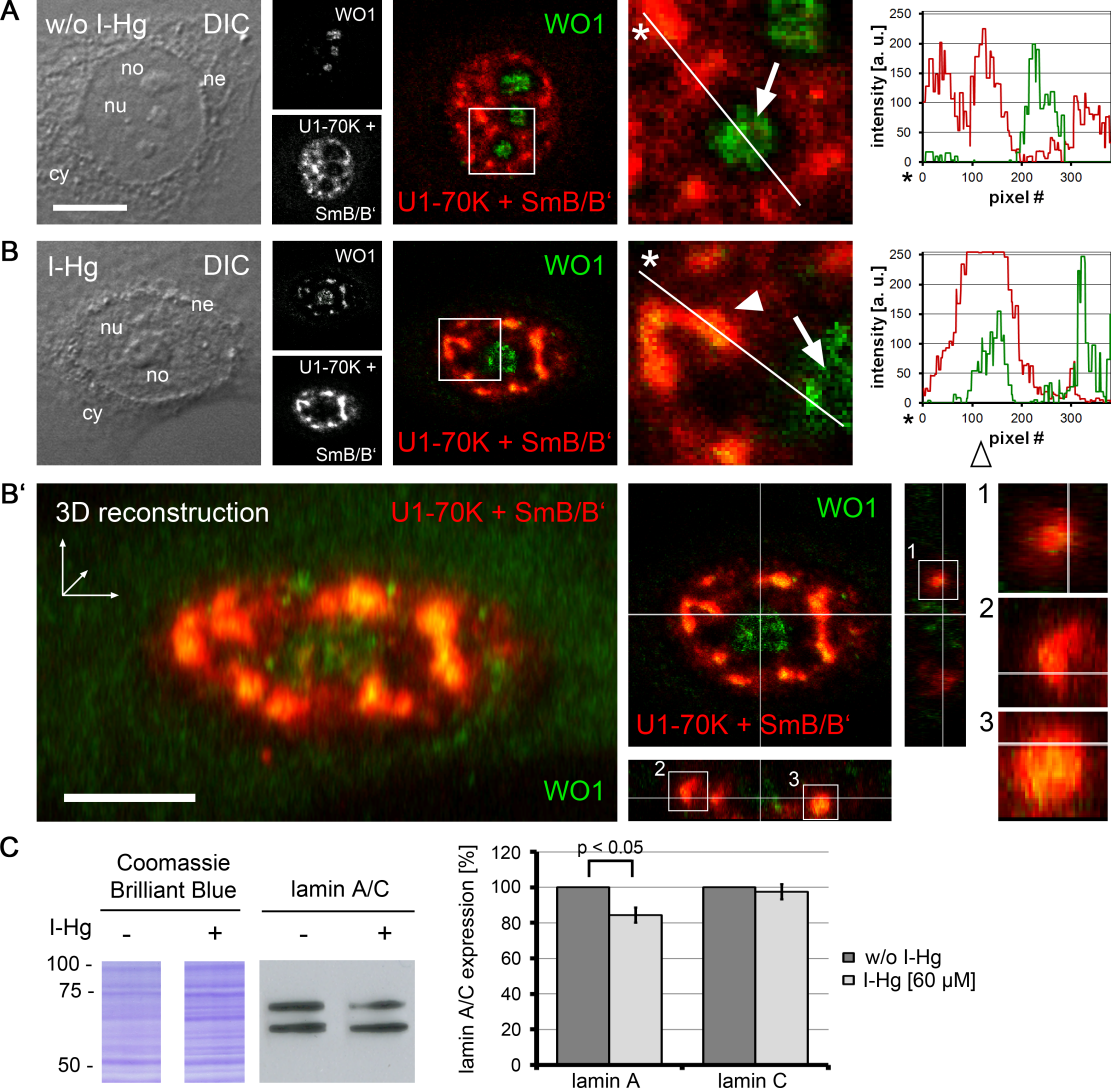
Amyloid-like microenvironments form in the center of nuclear speckles enriched with spliceosomal components. (A) Confocal immunofluorescence of WO1 (green) and spliceosomal components (human autoimmune serum against U1-70k and Sm-proteins, red) shows partial colocalization after (B) 4 h of I-Hg-treatment (merge, blow up). Separation or colocalization of WO1 and spliceosomal components is visualized by the linescan function of MetaMorph: the fluorescence intensity of each pixel of the line of interest (insets, asterisks, white lines) is shown as a *xy*-graph for the corresponding green and red channels. Arrows indicate nucleolar WO1-staining (green), whereas arrowheads indicate WO1-positive amyloid-like microenvironments in the nucleoplasm that colocalize with spliceosomal components (yellow). (B’) A *z*-scan of the cell shown in (B) indicates central positioning of WO1-labelled amyloid-like microenvironments within nuclear speckles. Confocal *xy*-planes of the *z*-stack were 3D-reconstructed by MetaMorph (B’, left). The same cell is shown by *z*-axis plane views along the indicated white lines, i.e., from the side and from below (B’, middle). Three exemplary aggregates are presented as blow ups of the *z*-axis-views (B’, right, insets 1–3). (C) shows a representative immunoblot of alternative splicing products lamin A/C in untreated (−) or I-Hg-treated (+) HEp-2 cells. Respective staining with the acid dye Coomassie brilliant blue indicates equal loading. The detected signals for lamin A/C were quantified by densitometric analysis, i.e., region of interest measurement using the software MetaMorph, and provided as mean values and standard deviations of three independent experiments. The values were normalized to the corresponding signal of untreated samples. A *p*-value < 0.05 indicates a statistical significant difference (tested with Student’s *t*-test). A.u., arbitrary units; cy, cytoplasm; DIC, differential interference contrast; h, hours; ne, nuclear envelope; no, nucleolus; nu, nucleus. Bars, 5 µm.

A prerequisite for protein–protein interactions is their subcellular localization. In order to investigate the location of I-Hg-induced nuclear amyloid in correlation to spliceosomes, confocal immunofluorescence was performed with antibodies that detect amyloid structures (Figs. 4A–4B’, WO1, green) and human antibodies that recognize spliceosomal components, i.e., U1-70k RNPs and Sm proteins (Figs. 4A–4B’, red). Confocal imaging shows that in ground state HEp-2 cells nuclear distribution of WO1 mainly occurs in nucleoli, whereas spliceosomal components U1-70K and Sm are distributed throughout the nucleoplasm and enriched in reticulated speckles (Fig. 4A, inset; compare with Fig. 2A). The respective line scan confirms that there is no overlap between amyloid structures in nucleoli and nucleoplasmic speckles. In an advanced fibrillation state, e.g., after induction with I-Hg, amyloid structures are not confined to nucleoli, but likewise occur in the nucleoplasm where they partially colocalize with spliceosomal components in reticulated speckles (Fig. 4B, inset, filled arrowhead, yellow). Colocalization of WO1 and U1-70k/SmB antibodies is depicted in the corresponding line scan (open arrowhead). The imaging results showing amyloid-like microenvironments in speckles enriched with spliceosomal components in I-Hg-treated HEp-2 cells (Fig. 4B, yellow) confirm mass spectrometry that identifies RNA processing factors as the major entity of the endogenous aggregome in an advanced fibrillation state due to the heavy metal Hg. Thus, in HEp-2 cells two fibrillation states are characterized by a transition from a ground state without detectable amyloid to an induced state where amyloid-like aggregation of spliceosomal components occurs in distinct microenvironments in the center (core) of nuclear speckles (Figs. 4B’, 3D reconstruction, *z*-axis views).

To investigate different protein fibrillation steps in correlation with RNA processing of specific targets, the ratio between the splice variants of lamin A/C was monitored in untreated and I-Hg-treated cells (Fig. 4C). Alternative splicing of the human *lmna* gene in exon 10 eventually gives rise to lamin A (69 kD) or lamin C (62 kD) proteins (Lin & Worman, 1993). Immunoblotting shows that induction of protein fibrillation by I-Hg is correlated with a significant reduction of lamin A expression by 20% (*p* < 0.05), whereas expression of lamin C remains unchanged (Fig. 4C, bar graph). This is consistent with the idea that amyloid fibrillation in nucleoplasmic microenvironments may modulate gene expression, here at the level of mRNA splicing.

### Nucleoskeletal protein lamin B1 regulates formation of speckles enriched with spliceosomal components and amyloid-like microenvironments

**Figure 5:**
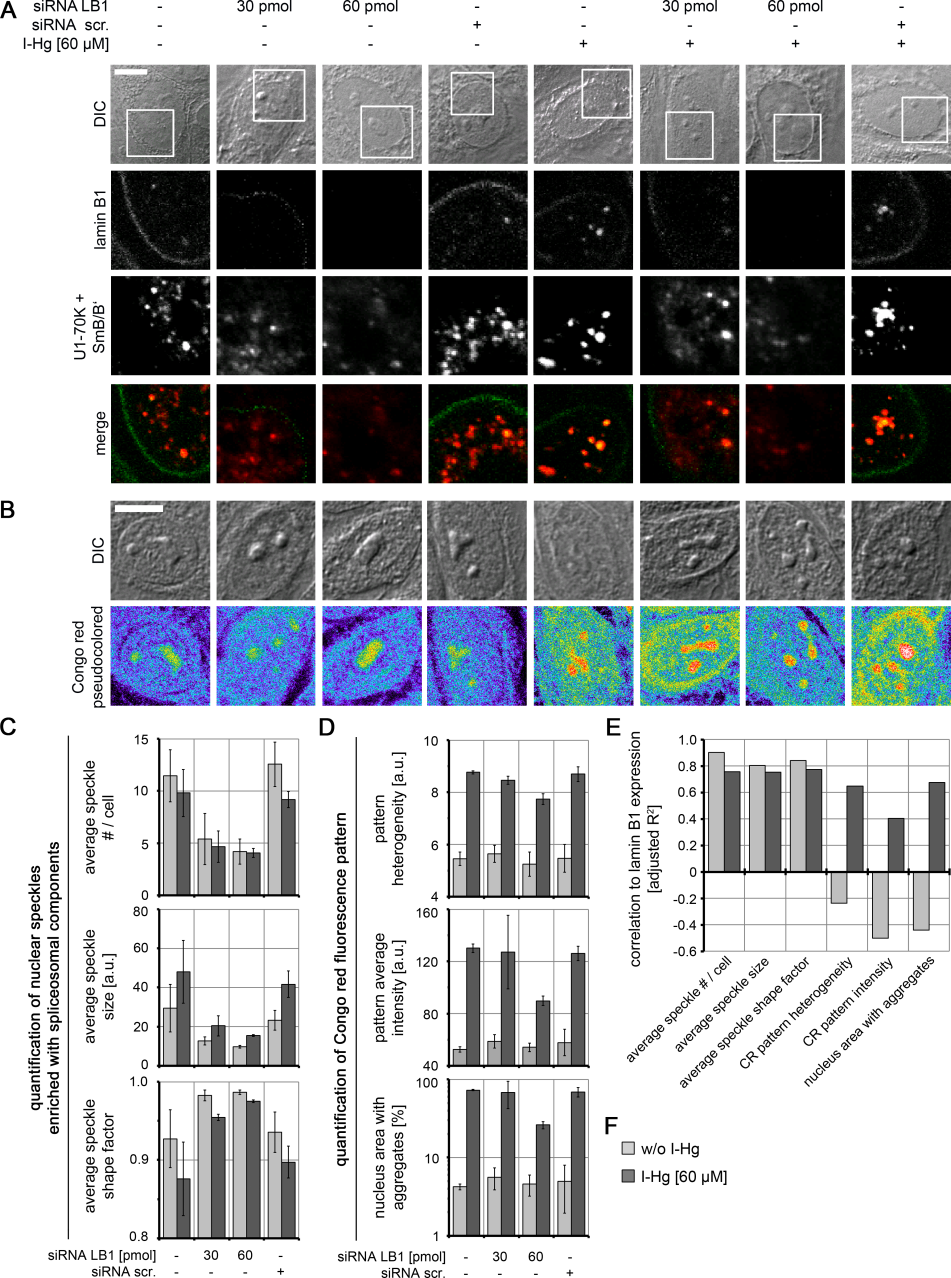
Nucleoskeletal protein lamin B1 regulates formation of I-Hg-induced nuclear amyloid. Increasing concentrations of short interfering RNAs (siRNAs, 30 pmol and 60 pmol) were used to deplete lamin B1 in untreated or I-Hg-treated HEp-2 cells. Reduced lamin B1 expression was confirmed by immunoblotting (Fig. S8). Controls include untreated or HEp-2 cells treated with scrambled, random siRNA (siRNA scr.). Cells were double-immunolabelled for lamin B1 and splicing components U1-70K/SmB/B’. (A) shows representative DICs of HEp-2 cells (top row) and indicated blow ups of respective nuclear envelope or nucleoplasmic regions with labelled lamin B1 (green) and U1-70K/SmB/B’ (red) in single fluorescence channels and merge. Yellow staining indicates colocalization of nucleoplasmic lamin B1 and nuclear speckles enriched with splicing components. (B) To investigate influence of lamin B1 depletion on amyloid formation, untreated or I-Hg-treated HEp-2 cells were stained for Congo red-positive microenvironments. Representative cells are shown in DIC and fluorescence channels (i.e., pseudocolored micrographs). Bars, 5 µm. (C) Immunostained nuclear speckles enriched with spliceosomal components were quantified using the Integrated Morphometry Analysis tool in Metamorph. Nuclear speckles were detected by intensity threshold and analysed for (i) average number of speckles per nucleus, (ii) average speckle size as (a.u.) and (iii) average speckle shape (shape factor = 1: round; shape factor < 1: reticulated). (D) shows quantification of the nuclear Congo red fluorescence pattern, as described previously (Arnhold & von Mikecz, 2011). The parameters for (i) Congo red pattern heterogeneity (a.u.), (ii) Congo red pattern intensity (a.u.) and (iii) nucleus area with Congo red positive aggregates (%) were used to quantitatively characterize nuclear protein aggregation/fibrillation. (C, D) show mean values and standard deviations of three independent experiments. (E) Correlation of lamin B1 depletion with nuclear speckle and Congo red pattern characteristics is calculated by linear fit analysis. Expression values of lamin B1 were determined by immunoblot analysis (shown in Fig. S8). Mean values of three independent experiments (presented in (C, D) and Fig. S8) were used to calculate correlations. The detailed correlation analysis is shown in Fig. S9 A.u., arbitrary units; I-Hg, inorganic mercury; LB1, lamin B1.

To understand the mechanism of the formation of amyloid-like microenvironments, the I-Hg-aggregome component lamin B1 was selected for further investigations, due to its participation in the nucleoskeleton (Simon & Wilson, 2011) and previously reported interactions with nuclear speckles enriched with spliceosomal components (Von Mikecz et al., 1997; Tang et al., 2008). HEp-2 cells were transfected with respective small interfering (si) RNAs in different concentrations to specifically knock down expression of lamin B1 (specificity of RNAi is shown in Fig. S8). By confocal immunofluorescence we observed in lamin B1 siRNA-treated cells a decrease of lamin B1 labelling at the nuclear envelope and in the nucleoplasm (Fig. 5A; green) as well as a reduced number of speckles enriched with spliceosomal components (Figs. 5A and 5C; red). This correlation between lamin B1 knock down and speckle formation was observed in untreated and I-Hg-treated cells, i.e., correlated with ground state as well as I-Hg-induced protein fibrillation (Fig. S9; Table S3).

RNAi experiments likewise demonstrate a correlation between reduction of lamin B1 expression and formation of Congo red-binding microenvironments that indicate nuclear amyloid (Fig. 5B). However, this correlation is particularly observed in I-Hg-treated cells (Figs. 5D–5F; Fig. S9; Table S4) suggesting that lamin B1 specifically sustains formation of amyloid-like microenvironments in the cell nucleus as part of the I-Hg-induced aggregome network (Table S1). This is consistent with the current view that lamin B1 forms a distinct filament network in living nuclei supporting nuclear processes such as transcription, replication and chromatin organization (Shimi et al., 2008; Simon & Wilson, 2011). Here, we introduce the idea that lamin B1 likewise regulates nuclear protein fibrillation, i.e., formation of nuclear amyloid.

## Conclusions

The present study identifies lamin B1-dependent amyloid formation in the cell nucleus as a novel bio-interaction of the global pollutant Hg. Similar to protein aggregation events in the cytoplasm that are controlled by the cytoskeleton (Kopito, 2000), the counterpart in the nucleus, e.g., amyloid fibrillation of nuclear proteins, may be driven by the nucleoskeleton. Characterization of the I-Hg-induced protein aggregation landscape reveals the nucleus as a major target of stepwise protein fibrillation that peaks with formation of amyloid microdomains in the core of speckles enriched with spliceosomal components. While substructures such as PML and Cajal nuclear bodies have previously been connected to neural nuclear inclusions in patients with CAG-repeat diseases and respective mouse models (Davies et al., 1997; Von Mikecz, 2014), speckles enriched with RNA processing factors are defined here as amyloidogenic venues and prevalent components of an endogenous aggregome for the first time. A notable similarity between transitional steps of amyloid fibrillation and formation of different spliceosomal complexes in the splicing cascade is partial structural unfolding of their protein components (Coelho Ribeiro Mde et al., 2013; Uversky, 2014). This similarity may explain the aggregation propensity of spliceosomal components and our observation of I-Hg-induced amyloid speckles. In fact, aggregome components such as FUS, hnRNPA1 or hnRNPA2/B1 contain prion like-domains with high prion scores, i.e., propensity for prion formation (Li et al., 2013). Accordingly, recruitment of FUS, hnRNPA1 and hnRNPA2/B1 to cytoplasmic stress granules was observed *in vitro* and in a subpopulation of patients with the progressive neurodegenerative disease amyotrophic lateral sclerosis (ALS) (Kim et al., 2013; Li et al., 2013). Although we do not detect accumulation of FUS or hnRNPs in cytoplasmic stress granules after I-Hg-treatment (data not shown), these proteins are clearly prone to undergo fibrillation in distinct subcellular domains that may depend on environmental conditions and the degree of protein fibrillation.

Components of the I-Hg-induced aggregome such as lamin B1, ubiquitin, heat shock protein HSP70 and spliceosomal aggregate components clearly participate in a framework of aberrant protein aggregation pathways in the nucleus. By identification of this aggregome network, we have just started to elucidate the interplay between nuclear protein homeostasis and I-Hg-induced impairment of neural function, e.g., neurotoxicity. As the extent of anthropogenic Hg release is becoming increasingly evident due to advanced global monitoring (Lamborg et al., 2014), and I-Hg rises in the US population in an age-dependent manner (Laks, 2014), our work urges further investigation of Hg-amyloid interactions in cross-species studies and translation of the results to epidemiologic data.

## Supplemental Information

10.7717/peerj.754/supp-1 Figure S1 Definition of sub-cytotoxic (‘mild’) I-Hg-concentrations Cell viability assay: HEp-2 or RA-differentiated SH-SY5Y cells were treated with the indicated I-Hg concentrations for 4 h and analyzed for intracellular staining of the diazo dye trypan blue designating dead cells. Results are presented as a *xy*-graph with the percentage of viable cells on the *y*-axis and the I-Hg-titration on the *x*-axis. Error bars represent standard deviation of three independent experiments. Arrows indicate the I-Hg-concentrations that do not induce cell death and were used in subsequent experiments to accelerate amyloid-like protein fibrillation in the nucleus.

10.7717/peerj.754/supp-2 Figure S2 Atomic absorption spectroscopy (AAS): protein fractionation controls Untreated or I-Hg-treated (4 h, 60 µM) HEp-2 cells were lysed and fractionated into cytoplasmic or nuclear proteins. Purity of fractions was controlled by immunoblots of (A) endoplasmatic reticulum-associated protein calnexin as cytoplasmic control or (B) spliceosomal component SmB/B’ as nuclear control. Respective Coomassie Brilliant Blue staining confirms equal protein loading. Expression levels of calnexin and SmB were quantified by densitometric analysis based on the band intensity of the immunoblots. a.u., arbitrary units; cy, cytoplasmic fraction; I-Hg, inorganic mercury; kDa, kilo Dalton; nu, nuclear fraction.

10.7717/peerj.754/supp-3 Figure S3 I-Hg induces aggregation of endogenous nuclear proteins in the nematode *C. elegans* One day-old, adult worms (wild-type N2) were left untreated (H_2_O control, upper panel) or treated with 60 µM I-Hg for 24 h (lower panel). Differential interference contrast and pseudocolored fluorescence microscopy micrographs of representative 2-day old worms are shown. Increasing intensities are depicted in purple (lowest intensity) via blue or red to white (highest intensity). White circles point out nuclei of the anterior-most intestinal cells that are shown in detail as blow ups (insets, right column)). DIC, differential interference contrast; I-Hg, inorganic mercury; nu, nucleus. Bar, 20 µm.

10.7717/peerj.754/supp-4 Figure S4 Validation of representative aggregome components (A) Untreated or I-Hg-treated SH-SY5Y neurons were analyzed by filter retardation assays. Dotblot immunodetection of filter-trapped SDS-insoluble protein aggregates with primary antibodies against beta tubulin, FUS/TLS, Hsc70, lamin B1, nucleolin (C23), nucleophosmin (B23), U1- 70K and SmB/B’ (spliceosomal components, human autoimmune serum), ubiquitin and CAG-repeats (polyQ). Experiments were carried out in triplicate with equal numbers of cells (3 × 10^6^ cells per dot). (B) Equal protein expression of representative aggregome components was controlled by immunoblotting of untreated and I-Hg-treated SH-SY5Y neurons (top). Respective staining of the SDS-PAGE-gel with the acid dye Coomassie Brilliant Blue indicates equal loading (bottom). h, hours; I-Hg, inorganic mercury.

10.7717/peerj.754/supp-5 Figure S5 I-Hg-induced recruitment of 20S proteasomes to amyloid speckles Representative confocal micrographs of (A) untreated or (B) I-Hg-treated (4h, 60 µM) HEp-2 cells, double-labelled for amyloid (WO1, green) and 20S proteasomes (red). Blow ups of indicated nuclear regions show WO1-positive nucleoli (arrows) and I-Hg-induced WO1-positive amyloid-like microenvironments in the nucleoplasm (filled arrowhead). Colocalization of amyloid-like microenvironments with proteasomes (yellow) is visualized in the corresponding linescan (open arrowhead). Bars, 5 µm.

10.7717/peerj.754/supp-6 Figure S6 I-Hg induces a significant increase of global proteasomal activity in the nucleus (A–C) HEp-2 cells were either left untreated or treated with I-Hg for 4 or 24 h followed by preparation of cytoplasmic and nuclear protein fractions. Cytoplasmic (A, A’) and nuclear (B, B’) fractions were analysed for proteasomal activity by incubation with fluorogenic substrate Suc-LLVY-AMC and measurement of fluorescence intensity for 960 min. Specificity of proteasomal degradation was tested by addition of proteasome inhibitor lactacystin after 2 h (light red, light green or light grey). (A’, B’) Bar graphs show mean values and standard deviations (SD) at time point *t* = 960 min (see A and B). One-way ANOVA with Tukey’s post-hoc test was performed to test for significant differences (*p* < 0.05). (C) Color codes indicate cell culture conditions. (D) Expression of 20S proteasomes was analysed by immunoblot of 20S alpha subunits. (E, F) show purity of cytoplasmic and nuclear protein fractions. Calnexin was used as a cytoplasmic marker and SmB/B’ was used as a nuclear marker. (D–F, bottom) Coomassie Brilliant Blue staining indicates equal protein loading. (A–F) Graphs show mean values of three independent experiments ±SD. AMC, aminomethylcoumarin; a.u., arbitrary units; cy, cytoplasm; DIC, differential interference contrast; h, hours; min, minutes; ne, nuclear envelope; no, nucleolus; nu, nucleus.

10.7717/peerj.754/supp-7 Figure S7 Protein features of aggregome components Protein bcomponents of aggregomes in different protein fibrillation states (compare Tables S1 and S2), i.e., HEp-2 ground state (black), HEp-2 I-Hg-induced (grey), SH-SY5Y ground state (red) and SH-SY5Y I-Hg-induced (green) were analyzed for sequence features extracted from the UniProtKB database. (A) The average number of features within each protein is calculated and presented as fold change to corresponding values of the complete human proteome database. The database reference line (*y*-value = 1) represents the value of a feature in the complete UniProtKB protein database. A corresponding value above 1 (upward bars) indicates statistical overrepresentation and a value below 1 (downward bars) indicates underrepresentation. Values of features that are not found in a single sample (value = 0) are not depicted. (B + B’) show the absolute values of the protein feature quantification described in (A). Additionally, the quantification of the complete protein database was plotted as a separate bar (blue). (B) The number of proteins with features (%) and (B’) the average number of features within a protein was calculated and is presented as bar plots of absolute values.

10.7717/peerj.754/supp-8 Figure S8 Expression of lamin B1 and spliceosomal component SmB/B’ in lamin B1 silenced cells HEp-2 cells were left untreated, or pre-treated with lamin B1 siRNA (30 pmol or 60 pmol) or scrambled siRNA, followed by an incubation with I-Hg (4 h, (60 µM)) as indicated. Cell lysates were analysed by immunoblotting and expression levels of (A) SmB/B’ and (B) lamin B1 were quantified by densitometric analysis. Coomassie Brilliant Blue staining indicates equal loading. Immunoblots are representative of three independent experiments. A.u., arbitrary units; kDA, kilo Dalton; LB1, lamin B1; siRNA scr., scrambled small interfering RNA.

10.7717/peerj.754/supp-9 Figure S9 Positive correlation of lamin B1 depletion with I-Hg-induced nuclear amyloid Correlation analysis was done by linear fit analysis (Origin 8.5, Origin Labs). High adjusted *R*^2^-values and corresponding high eccentricity of the confidence ellipses indicate a high correlation. A positive or negative slope of the regression line indicates a positive or negative correlation. Adjusted *R*^2^-values were used to evaluate correlation of lamin B1 expression levels with speckle and amyloid fluorescence pattern characteristics. Scatter plots show lamin B1 expression (immunoblot) on the *x*-axis and speckle or amyloid characteristics (immunofluorescence) on the *y* axis. Graphs show mean values (X) from quantifications in Figs. 5C and 5D, linear regression (line), confidence ellipse (ellipse) and adjusted *R*^2^-values (text). The analysis shows a high correlation of nuclear speckle patterns with expression of lamin B1 in untreated as well as in I-Hg-treated cells. Nuclear speckles enriched with spliceosomal components are reduced in number and size and become rounder when lamin B1 is depleted by RNA interference (compare to Fig. 5). The ground state of nuclear protein aggregation (Congo red pattern in untreated cells) is not correlated with lamin B1 depletion, as indicated by low *R*^2^-values and round shaped confidence ellipses (eccentricity near 0). In contrast, lamin B1 depletion is positively correlated with I-Hg-induced nuclear amyloid as indicated by high eccentricity and positive slopes of the respective confidence ellipses. The data suggests a critical role of lamin B1 in nuclear speckle formation and induction of nuclear amyloid. adj, adjusted; h, hours; I-Hg, inorganic mercury; *R*^2^, coefficient of determination.

10.7717/peerj.754/supp-10 Table S1 The aggregome of untreated or I-Hg-induced HEp-2 cells as identified by mass spectrometric analysis Table S1 lists all filter-trapped proteins detected in untreated HEp-2 cell samples (ground state protein fibrillation) and in samples from 4 h I-Hg-treated HEp-2 cells (induced fibrillation state), depleted of candidates that also occur in ground state. Proteins from two independent experiments per group were pooled and listed. Entry names are according to the SwissProt database.

10.7717/peerj.754/supp-11 Table S2 The aggregome of untreated or I-Hg-induced neural SHSY5Y cells as identified by mass spectrometric analysis Table S2 lists all filter-trapped proteins detected in untreated SH-SY5Y cell samples (ground state protein fibrillation) and in samples from 4 h I-Hg-treated SHSY5Y cells (induced fibrillation state), depleted of candidates that also occur in ground state. Proteins from two independent experiments per group were pooled and listed. Entry names are according to the SwissProt database.

10.7717/peerj.754/supp-12 Table S3 Statistical analysis of the quantification of nuclear speckle patterns (compare Figs. 5A and 5C) Mean values presented in Fig. 5C were tested for significance by one-way ANOVA and Tukey’s post-hoc test. Values indicating significance (*p* < 0.05) are depicted in black.

10.7717/peerj.754/supp-13 Table S4 Statistical analysis of the quantification of nuclear Congo red staining patterns (compare Figs. 5B and 5D) Mean values from Fig. 5D were tested for significance by one-way ANOVA and Tukey’s post-hoc test. Values indicating significance (*p* < 0.05) are depicted in bold, black lettering.

10.7717/peerj.754/supp-14 Data S1 Raw data of Fluo4 measurements in Fig. 1A–1D.

10.7717/peerj.754/supp-15 Data S2 Raw data of aggregome proteomics Raw data of mass spectrometry results, e.g., aggregome components in Tables S1 and S2.

10.7717/peerj.754/supp-16 Data S3 Raw data of Fig. S1
Raw data of cell viability analyses in HEp-2 or SH-SY5Y cells that were treated with increasing concentrations of I-Hg as presented in Fig. S1.

10.7717/peerj.754/supp-17 Data S4 Raw data of proteasomal activity analyses Raw data of global proteasomal activity in cytoplasmic or nuclear protein fractions of untreated or I-Hg-treated HEp-2 cells as presented in Figs. S6A, S6A’, S6B, S6B’.

10.7717/peerj.754/supp-18 Data S5 Raw data of immunoblots (Figs. 1, 4, Figs. S2, S4, S6, S8).
