# Supplementary figures and images for: Amyloid domains in the cell nucleus controlled by nucleoskeletal protein lamin B1 reveal a new pathway of mercury neurotoxicity
Source: PeerJ. 2015 Feb 5;3:e754. doi: 10.7717/peerj.754 (PMC4327309; doi:10.7717/peerj.754)

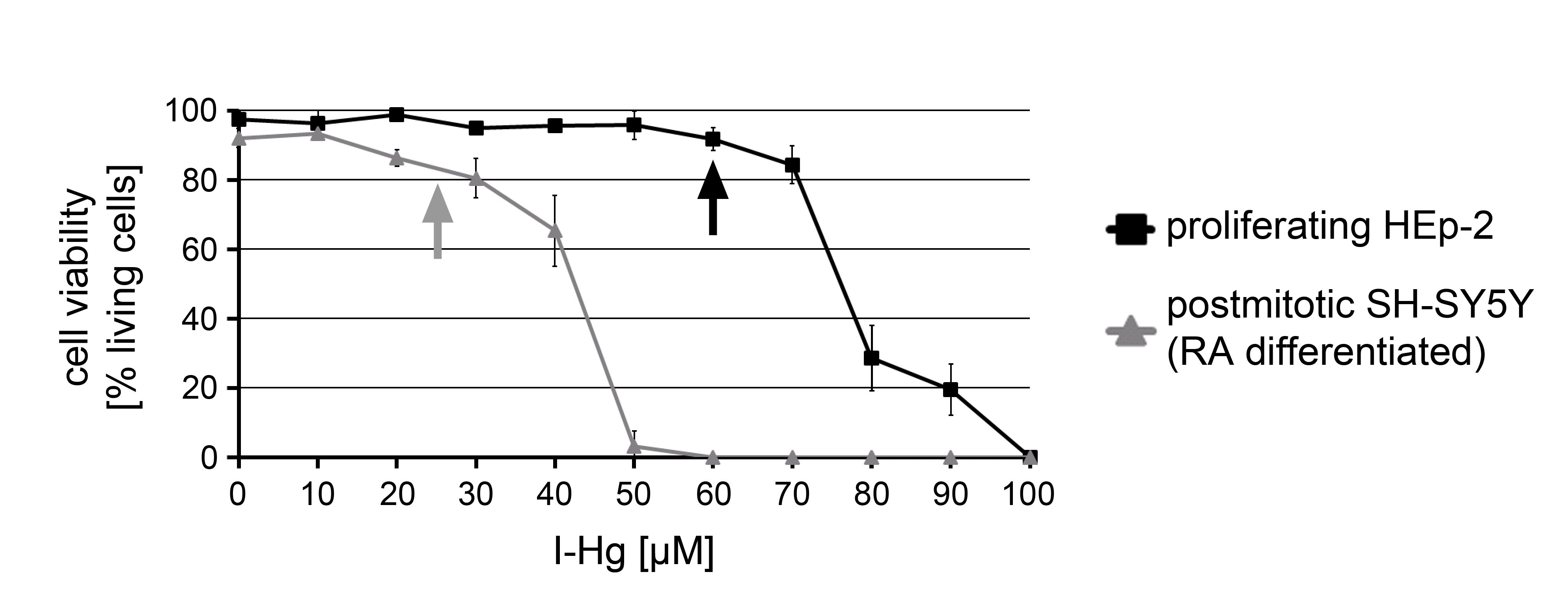

Supplement: Figure S1 — Cell viability assay: HEp-2 or RA-differentiated SH-SY5Y cells were treated with the indicated I-Hg concentrations for 4 h and analyzed for intracellular staining of the diazo dye trypan blue designating dead cells. Results are presented as a xy-graph with the percentage of viable cells on the y-axis and the I-Hg-titration on the x-axis. Error bars represent standard deviation of three independent experiments. Arrows indicate the I-Hg-concentrations that do not induce cell death and were used in subsequent experiments to accelerate amyloid-like protein fibrillation in the nucleus. [file peerj-03-754-s001.png]

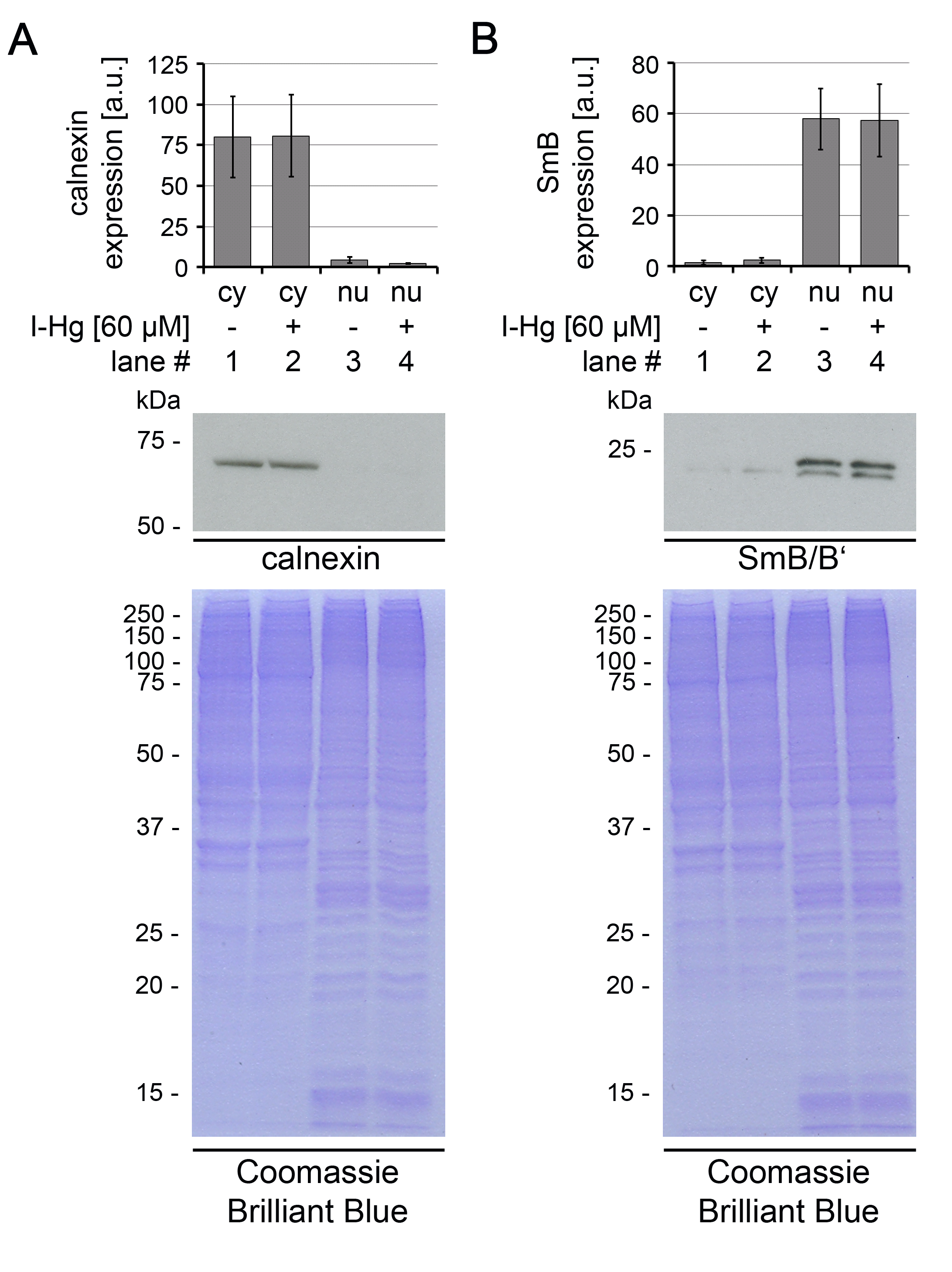

Supplement: Figure S2 — Untreated or I-Hg-treated (4 h, 60 µM) HEp-2 cells were lysed and fractionated into cytoplasmic or nuclear proteins. Purity of fractions was controlled by immunoblots of (A) endoplasmatic reticulum-associated protein calnexin as cytoplasmic control or (B) spliceosomal component SmB/B’ as nuclear control. Respective Coomassie Brilliant Blue staining confirms equal protein loading. Expression levels of calnexin and SmB were quantified by densitometric analysis based on the band intensity of the immunoblots. a.u., arbitrary units; cy, cytoplasmic fraction; I-Hg, inorganic mercury; kDa, kilo Dalton; nu, nuclear fraction. [file peerj-03-754-s002.png]

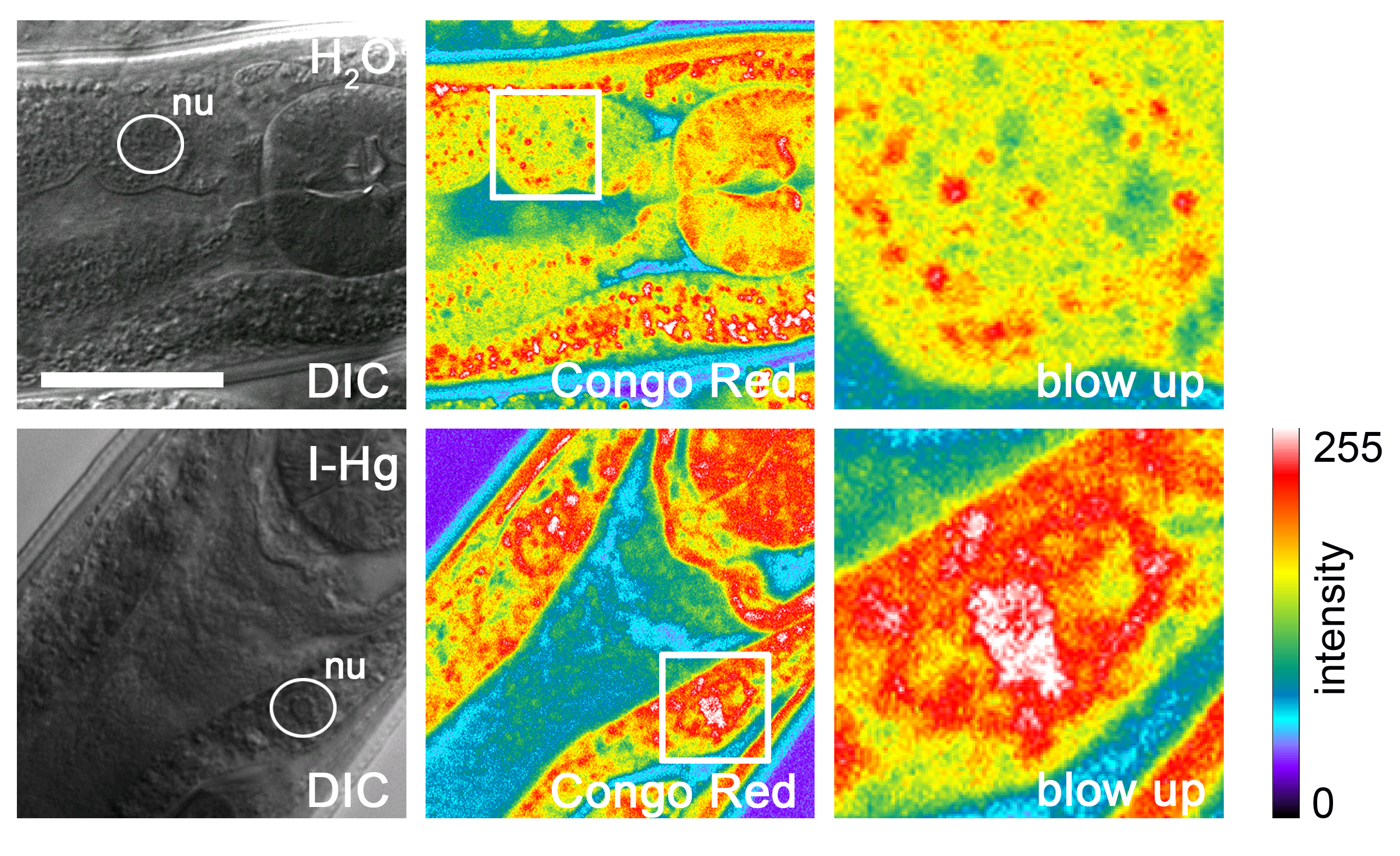

Supplement: Figure S3 — One day-old, adult worms (wild-type N2) were left untreated (H2O control, upper panel) or treated with 60 µM I-Hg for 24 h (lower panel). Differential interference contrast and pseudocolored fluorescence microscopy micrographs of representative 2-day old worms are shown. Increasing intensities are depicted in purple (lowest intensity) via blue or red to white (highest intensity). White circles point out nuclei of the anterior-most intestinal cells that are shown in detail as blow ups (insets, right column)). DIC, differential interference contrast; I-Hg, inorganic mercury; nu, nucleus. Bar, 20 µm. [file peerj-03-754-s003.png]

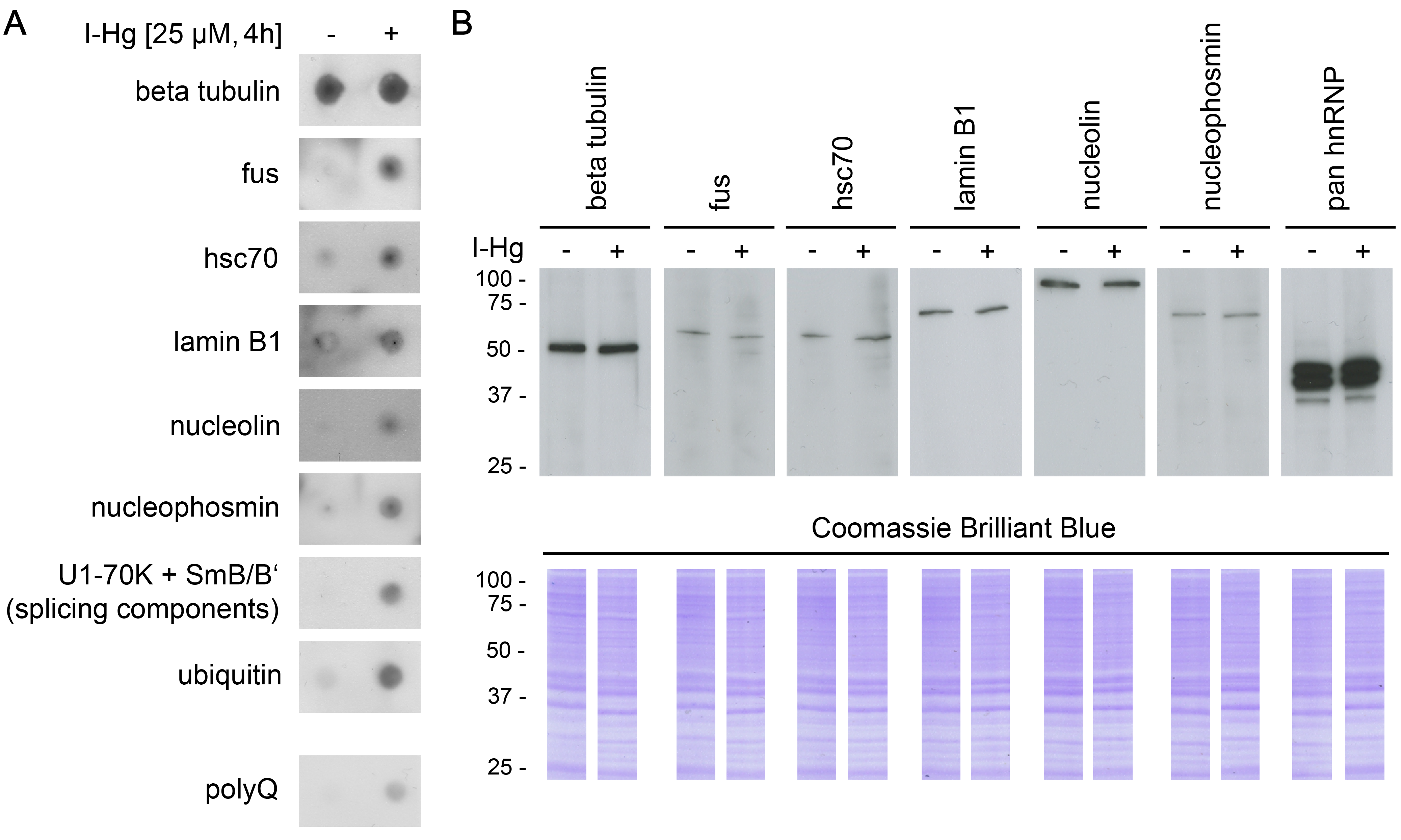

Supplement: Figure S4 — (A) Untreated or I-Hg-treated SH-SY5Y neurons were analyzed by filter retardation assays. Dotblot immunodetection of filter-trapped SDS-insoluble protein aggregates with primary antibodies against beta tubulin, FUS/TLS, Hsc70, lamin B1, nucleolin (C23), nucleophosmin (B23), U1- 70K and SmB/B’ (spliceosomal components, human autoimmune serum), ubiquitin and CAG-repeats (polyQ). Experiments were carried out in triplicate with equal numbers of cells (3 × 10^6^ cells per dot). (B) Equal protein expression of representative aggregome components was controlled by immunoblotting of untreated and I-Hg-treated SH-SY5Y neurons (top). Respective staining of the SDS-PAGE-gel with the acid dye Coomassie Brilliant Blue indicates equal loading (bottom). h, hours; I-Hg, inorganic mercury. [file peerj-03-754-s004.png]

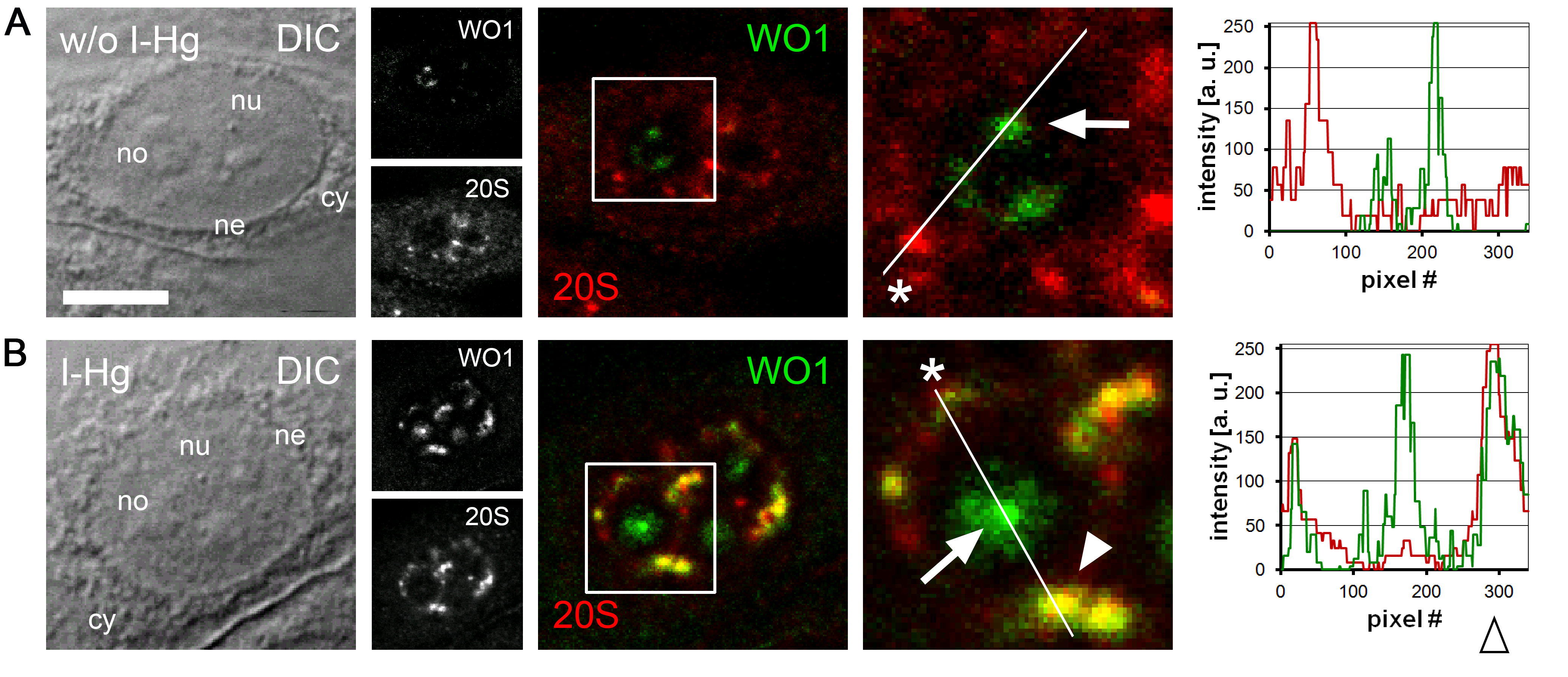

Supplement: Figure S5 — Representative confocal micrographs of (A) untreated or (B) I-Hg-treated (4h, 60 µM) HEp-2 cells, double-labelled for amyloid (WO1, green) and 20S proteasomes (red). Blow ups of indicated nuclear regions show WO1-positive nucleoli (arrows) and I-Hg-induced WO1-positive amyloid-like microenvironments in the nucleoplasm (filled arrowhead). Colocalization of amyloid-like microenvironments with proteasomes (yellow) is visualized in the corresponding linescan (open arrowhead). Bars, 5 µm. [file peerj-03-754-s005.png]

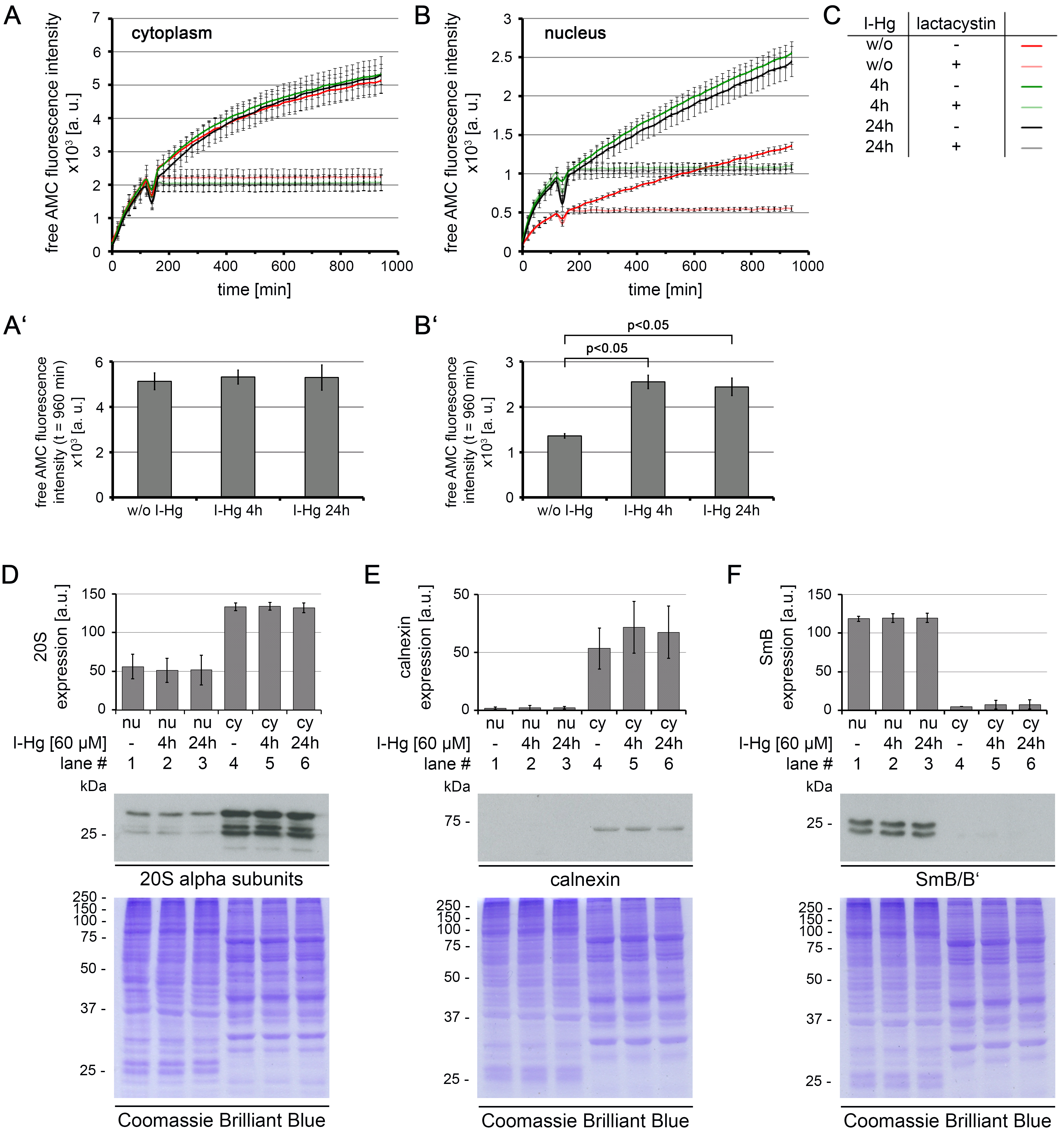

Supplement: Figure S6 — (A–C) HEp-2 cells were either left untreated or treated with I-Hg for 4 or 24 h followed by preparation of cytoplasmic and nuclear protein fractions. Cytoplasmic (A, A’) and nuclear (B, B’) fractions were analysed for proteasomal activity by incubation with fluorogenic substrate Suc-LLVY-AMC and measurement of fluorescence intensity for 960 min. Specificity of proteasomal degradation was tested by addition of proteasome inhibitor lactacystin after 2 h (light red, light green or light grey). (A’, B’) Bar graphs show mean values and standard deviations (SD) at time point t = 960 min (see A and B). One-way ANOVA with Tukey’s post-hoc test was performed to test for significant differences (p < 0.05). (C) Color codes indicate cell culture conditions. (D) Expression of 20S proteasomes was analysed by immunoblot of 20S alpha subunits. (E, F) show purity of cytoplasmic and nuclear protein fractions. Calnexin was used as a cytoplasmic marker and SmB/B’ was used as a nuclear marker. (D–F, bottom) Coomassie Brilliant Blue staining indicates equal protein loading. (A–F) Graphs show mean values of three independent experiments ±SD. AMC, aminomethylcoumarin; a.u., arbitrary units; cy, cytoplasm; DIC, differential interference contrast; h, hours; min, minutes; ne, nuclear envelope; no, nucleolus; nu, nucleus. [file peerj-03-754-s006.png]

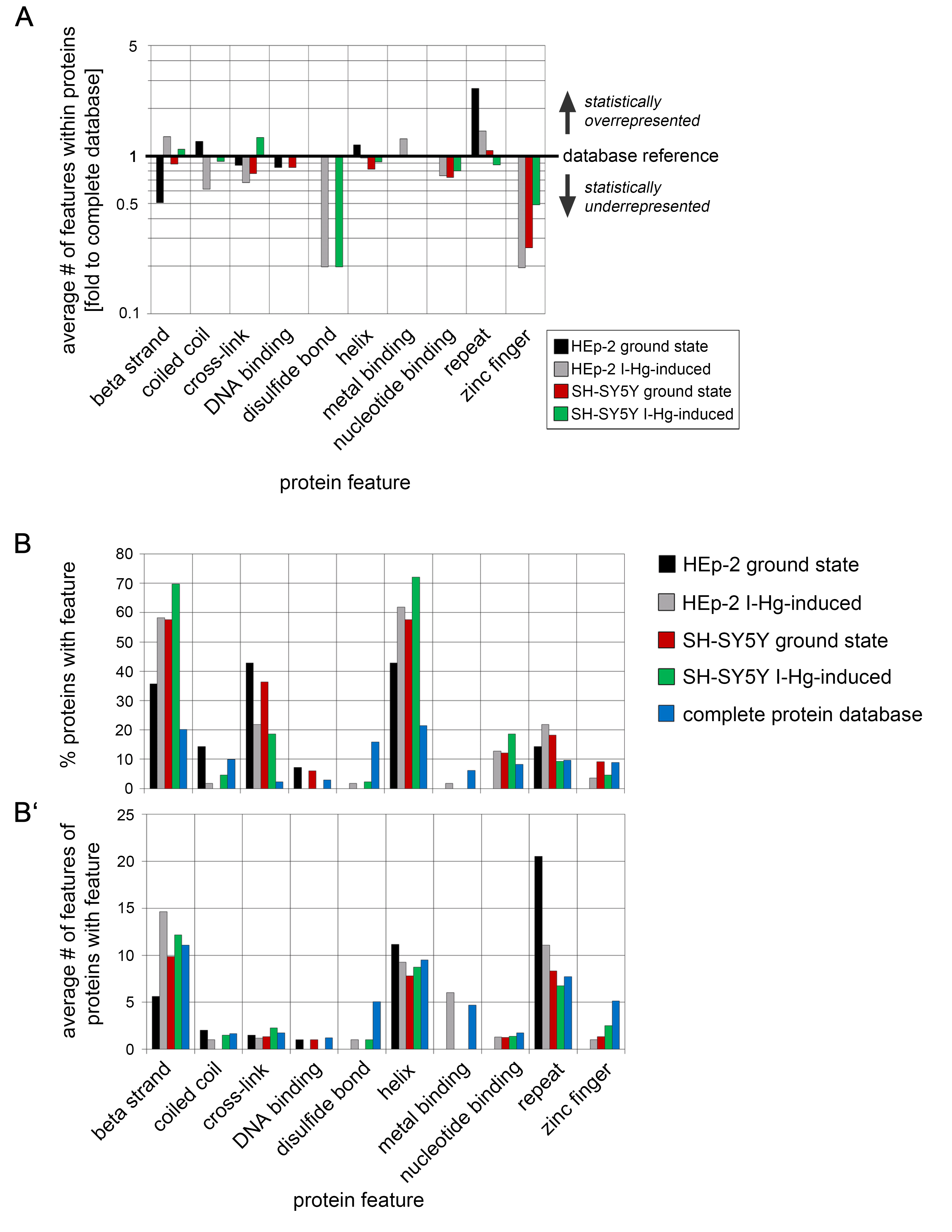

Supplement: Figure S7 — Protein bcomponents of aggregomes in different protein fibrillation states (compare Tables S1 and S2), i.e., HEp-2 ground state (black), HEp-2 I-Hg-induced (grey), SH-SY5Y ground state (red) and SH-SY5Y I-Hg-induced (green) were analyzed for sequence features extracted from the UniProtKB database. (A) The average number of features within each protein is calculated and presented as fold change to corresponding values of the complete human proteome database. The database reference line (y-value = 1) represents the value of a feature in the complete UniProtKB protein database. A corresponding value above 1 (upward bars) indicates statistical overrepresentation and a value below 1 (downward bars) indicates underrepresentation. Values of features that are not found in a single sample (value = 0) are not depicted. (B + B’) show the absolute values of the protein feature quantification described in (A). Additionally, the quantification of the complete protein database was plotted as a separate bar (blue). (B) The number of proteins with features (%) and (B’) the average number of features within a protein was calculated and is presented as bar plots of absolute values. [file peerj-03-754-s007.png]

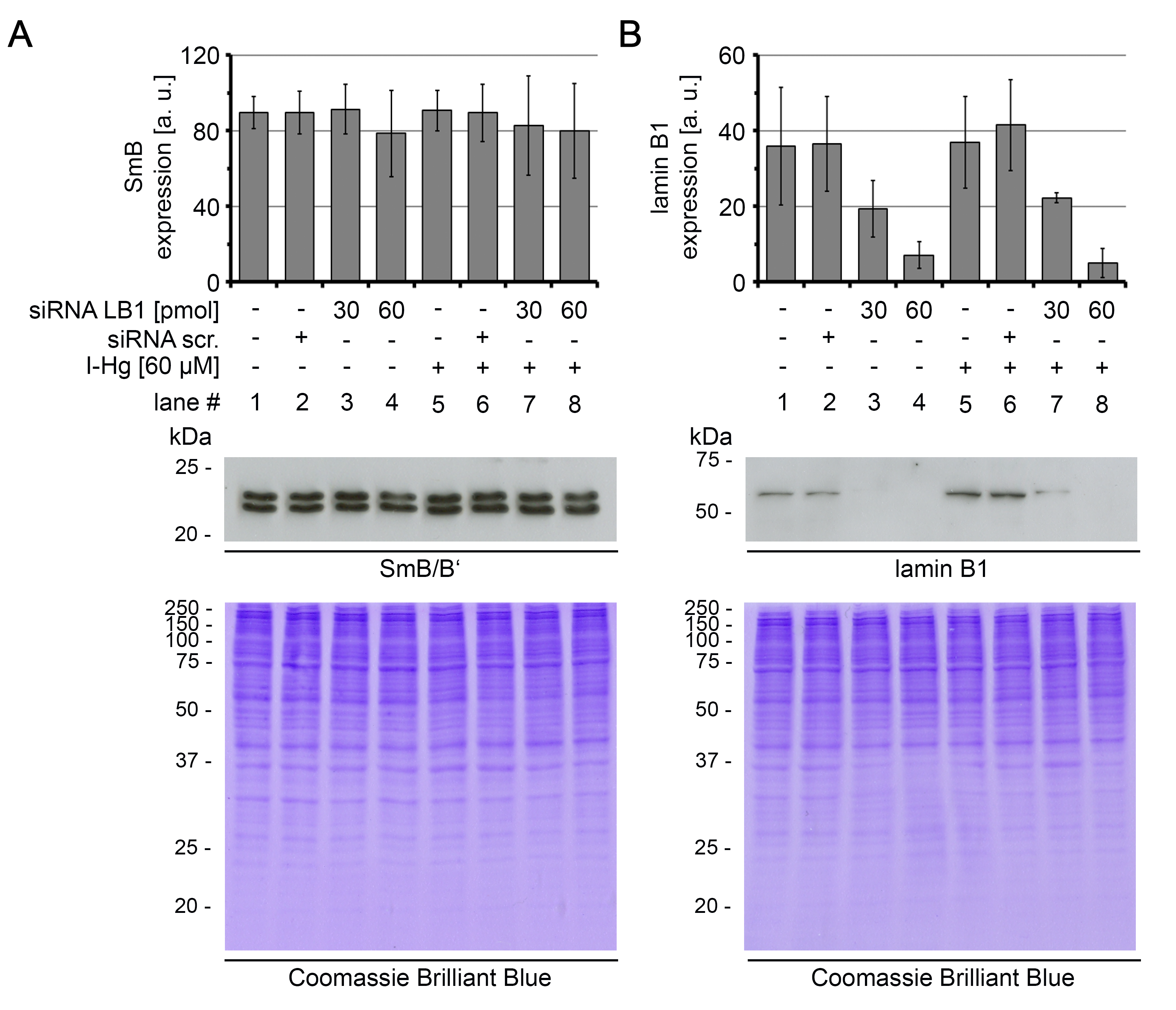

Supplement: Figure S8 — HEp-2 cells were left untreated, or pre-treated with lamin B1 siRNA (30 pmol or 60 pmol) or scrambled siRNA, followed by an incubation with I-Hg (4 h, (60 µM)) as indicated. Cell lysates were analysed by immunoblotting and expression levels of (A) SmB/B’ and (B) lamin B1 were quantified by densitometric analysis. Coomassie Brilliant Blue staining indicates equal loading. Immunoblots are representative of three independent experiments. A.u., arbitrary units; kDA, kilo Dalton; LB1, lamin B1; siRNA scr., scrambled small interfering RNA. [file peerj-03-754-s008.png]

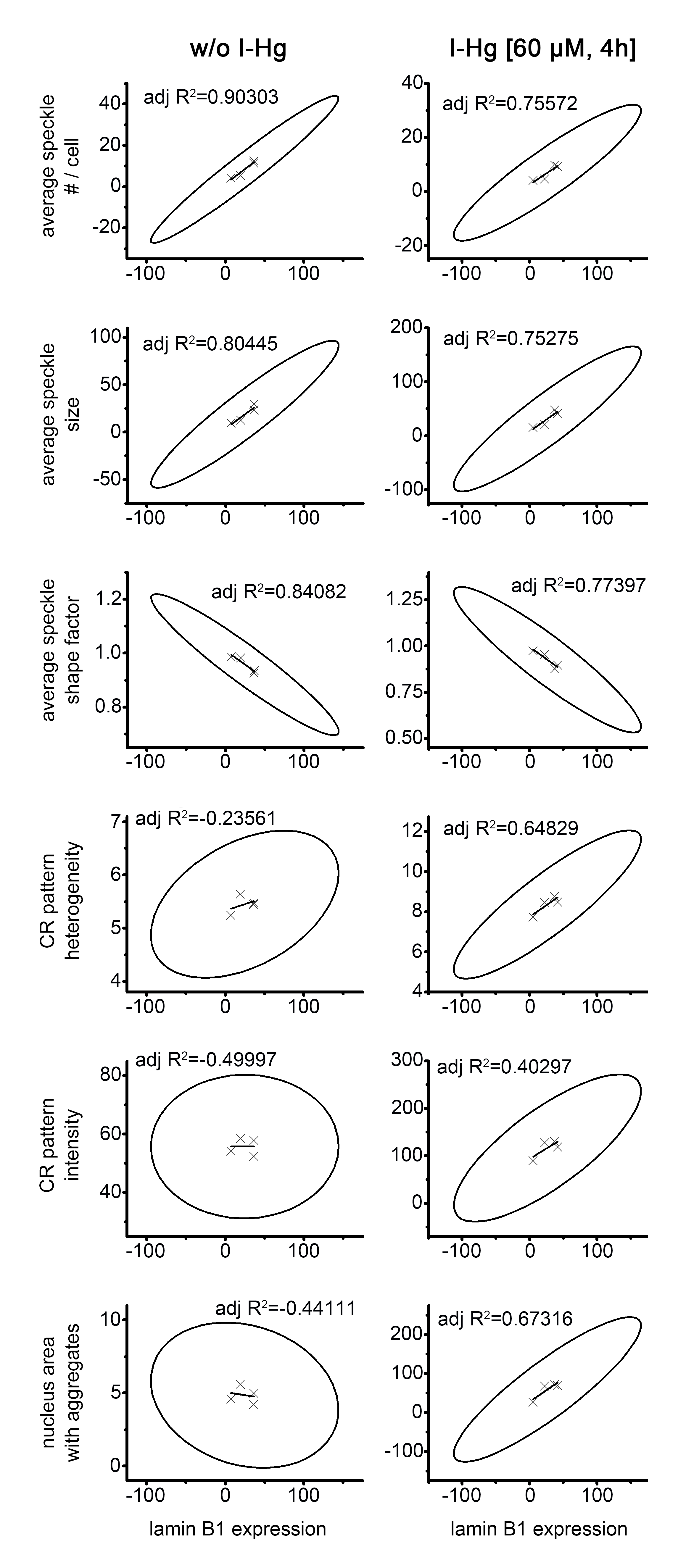

Supplement: Figure S9 — Correlation analysis was done by linear fit analysis (Origin 8.5, Origin Labs). High adjusted R2-values and corresponding high eccentricity of the confidence ellipses indicate a high correlation. A positive or negative slope of the regression line indicates a positive or negative correlation. Adjusted R2-values were used to evaluate correlation of lamin B1 expression levels with speckle and amyloid fluorescence pattern characteristics. Scatter plots show lamin B1 expression (immunoblot) on the x-axis and speckle or amyloid characteristics (immunofluorescence) on the y axis. Graphs show mean values (X) from quantifications in Figs. 5C and 5D, linear regression (line), confidence ellipse (ellipse) and adjusted R2-values (text). The analysis shows a high correlation of nuclear speckle patterns with expression of lamin B1 in untreated as well as in I-Hg-treated cells. Nuclear speckles enriched with spliceosomal components are reduced in number and size and become rounder when lamin B1 is depleted by RNA interference (compare to Fig. 5). The ground state of nuclear protein aggregation (Congo red pattern in untreated cells) is not correlated with lamin B1 depletion, as indicated by low R2-values and round shaped confidence ellipses (eccentricity near 0). In contrast, lamin B1 depletion is positively correlated with I-Hg-induced nuclear amyloid as indicated by high eccentricity and positive slopes of the respective confidence ellipses. The data suggests a critical role of lamin B1 in nuclear speckle formation and induction of nuclear amyloid. adj, adjusted; h, hours; I-Hg, inorganic mercury; R2, coefficient of determination. [file peerj-03-754-s009.png]
