# Supplementary material for: Amyloid domains in the cell nucleus controlled by nucleoskeletal protein lamin B1 reveal a new pathway of mercury neurotoxicity
Source: PeerJ. 2015 Feb 5;3:e754. doi: 10.7717/peerj.754 (PMC4327309; doi:10.7717/peerj.754)
Supplement: Table S1 — lists all filter-trapped proteins detected in untreated HEp-2 cell samples (ground state protein fibrillation) and in samples from 4 h I-Hg-treated HEp-2 cells (induced fibrillation state), depleted of candidates that also occur in ground state. Proteins from two independent experiments per group were pooled and listed. Entry names are according to the SwissProt database. [file peerj-03-754-s010.docx]

**Supplemental Table S1.** The aggregome of untreated or I-Hg-induced HEp-2 cells as identified by mass spectrometric analysis.

| **HEp-2 ground state** | |  | **HEp-2 4h I-Hg-induced** | |
| --- | --- | --- | --- | --- |
| **entry name** | **protein name** |  | **entry name** | **protein name** |
| ACTB_HUMAN | Actin, cytoplasmic 1 |  | ANXA2_HUMAN | Annexin A2 |
| ACTN4_HUMAN | Alpha-actinin-4 |  | CH60_HUMAN | 60 kDa heat shock protein, mitochondrial |
| CBX3_HUMAN | Chromobox protein homolog 3 |  | DDX21_HUMAN | Nucleolar RNA helicase 2 |
| H12_HUMAN | Histone H1.2 |  | DDX3X_HUMAN | ATP-dependent RNA helicase DDX3X |
| H2A2A_HUMAN | Histone H2A type 2-A |  | DDX5_HUMAN | Probable ATP-dependent RNA helicase DDX5 |
| H2B1H_HUMAN | Histone H2B type 1-H |  | FLNA_HUMAN | Filamin-A |
| H4_HUMAN | Histone H4 |  | FLNB_HUMAN | Filamin-B |
| K1C17_HUMAN | Keratin, type I cytoskeletal 17 |  | FUS_HUMAN | RNA-binding protein FUS |
| K1C9_HUMAN | Keratin, type I cytoskeletal 9 |  | FUSIP_HUMAN | FUS-interacting serine-arginine-rich protein 1 |
| K2C1_HUMAN | Keratin, type II cytoskeletal 1 |  | G3P_HUMAN | Glyceraldehyde-3-phosphate dehydrogenase |
| K2C8_HUMAN | Keratin, type II cytoskeletal 8 |  | GRP75_HUMAN | Stress-70 protein, mitochondrial |
| LMNA_HUMAN | Lamin-A/C |  | H15_HUMAN | Histone H1.5 |
| PLEC1_HUMAN | Plectin-1 |  | H2AV_HUMAN | Histone H2A.V |
| VIME_HUMAN | Vimentin |  | H2B1B_HUMAN | Histone H2B type 1-B |
|  |  |  | H2B1D_HUMAN | Histone H2B type 1-D |
|  |  |  | H31_HUMAN | Histone H3.1 |
|  |  |  | H33_HUMAN | Histone H3.3 |
|  |  |  | HNRDL_HUMAN | Heterogeneous nuclear ribonucleoprotein D-like |
|  |  |  | HNRH1_HUMAN | Heterogeneous nuclear ribonucleoprotein H |
|  |  |  | HNRH3_HUMAN | Heterogeneous nuclear ribonucleoprotein H3 |
|  |  |  | HNRPC_HUMAN | Heterogeneous nuclear ribonucleoproteins C1/C2 |
|  |  |  | HNRPG_HUMAN | Processed heterogeneous nuclear ribonucleoprotein G |
|  |  |  | HNRPK_HUMAN | Heterogeneous nuclear ribonucleoprotein K |
|  |  |  | HNRPL_HUMAN | Heterogeneous nuclear ribonucleoprotein L |
|  |  |  | HNRPQ_HUMAN | Heterogeneous nuclear ribonucleoprotein Q |
|  |  |  | HNRPR_HUMAN | Heterogeneous nuclear ribonucleoprotein R |
|  |  |  | HP1B3_HUMAN | Heterochromatin protein 1-binding protein 3 |
|  |  |  | HSP7C_HUMAN | Heat shock cognate 71 kDa protein |
|  |  |  | K1C18_HUMAN | Keratin, type I cytoskeletal 18 |
|  |  |  | K2C7_HUMAN | Keratin, type II cytoskeletal 7 |
|  |  |  | KPYM_HUMAN | Pyruvate kinase isozymes M1/M2 |
|  |  |  | LMNB1_HUMAN | Lamin-B1 |
|  |  |  | MATR3_HUMAN | Matrin-3 |
|  |  |  | MYH9_HUMAN | Myosin-9 |
|  |  |  | NPM_HUMAN | Nucleophosmin |
|  |  |  | NUCL_HUMAN | Nucleolin |
|  |  |  | PGK1_HUMAN | Phosphoglycerate kinase 1 |
|  |  |  | PPIA_HUMAN | Peptidyl-prolyl cis-trans isomerase A |
|  |  |  | RL18_HUMAN | 60S ribosomal protein L18 |
|  |  |  | RL4_HUMAN | 60S ribosomal protein L4 |
|  |  |  | RLA1_HUMAN | 60S acidic ribosomal protein P1 |
|  |  |  | ROA1_HUMAN | Heterogeneous nuclear ribonucleoprotein A1 |
|  |  |  | ROA2_HUMAN | Heterogeneous nuclear ribonucleoproteins A2/B1 |
|  |  |  | RRS1_HUMAN | Ribosome biogenesis regulatory protein homolog |
|  |  |  | RS5_HUMAN | 40S ribosomal protein S5 |
|  |  |  | RS7_HUMAN | 40S ribosomal protein S7 |
|  |  |  | SFPQ_HUMAN | Splicing factor, proline- and glutamine-rich |
|  |  |  | SFRS1_HUMAN | Splicing factor, arginine/serine-rich 1 |
|  |  |  | SFRS2_HUMAN | Splicing factor, arginine/serine-rich 2 |
|  |  |  | SPTA2_HUMAN | Spectrin alpha chain, brain |
|  |  |  | SPTB2_HUMAN | Spectrin beta chain, brain 1 |
|  |  |  | TBB5_HUMAN | Tubulin beta chain |
|  |  |  | TCP4_HUMAN | Activated RNA polymerase II transcriptional coactivator p15 |
|  |  |  | THOC4_HUMAN | THO complex subunit 4 |
|  |  |  | TPIS_HUMAN | Triosephosphate isomerase |
