# Supplementary material for: Amyloid domains in the cell nucleus controlled by nucleoskeletal protein lamin B1 reveal a new pathway of mercury neurotoxicity
Source: PeerJ. 2015 Feb 5;3:e754. doi: 10.7717/peerj.754 (PMC4327309; doi:10.7717/peerj.754)
Supplement: Table S2 — lists all filter-trapped proteins detected in untreated SH-SY5Y cell samples (ground state protein fibrillation) and in samples from 4 h I-Hg-treated SHSY5Y cells (induced fibrillation state), depleted of candidates that also occur in ground state. Proteins from two independent experiments per group were pooled and listed. Entry names are according to the SwissProt database. [file peerj-03-754-s011.docx]

**Supplemental Table S2.** The aggregome of untreated or I-Hg-induced neural SHSY5Y cells as identified by mass spectrometric analysis.

| **SH-SY5Y ground state** | |  | **SH-SY5Y 4h I-Hg-induced** | |
| --- | --- | --- | --- | --- |
| **entry name** | **protein name** |  | **entry name** | **protein name** |
| CBX1_HUMAN | Chromobox protein homolog 1 |  | ACTB_HUMAN | Actin, cytoplasmic 1 |
| DDX5_HUMAN | Probable ATP-dependent RNA helicase DDX5 |  | CBX3_HUMAN | Chromobox protein homolog 3 |
| H12_HUMAN | Histone H1.2 |  | DDX3X_HUMAN | ATP-dependent RNA helicase DDX3X |
| H15_HUMAN | Histone H1.5 |  | DHX9_HUMAN | ATP-dependent RNA helicase A |
| H2A2A_HUMAN | Histone H2A type 2-A |  | EF1A1_HUMAN | Elongation factor 1-alpha 1 |
| H2AV_HUMAN | Histone H2A.V |  | ELAV1_HUMAN | ELAV-like protein 1 |
| H2B1D_HUMAN | Histone H2B type 1-D |  | FUBP1_HUMAN | Far upstream element-binding protein 1 |
| H2B1H_HUMAN | Histone H2B type 1-H |  | FUBP2_HUMAN | Far upstream element-binding protein 2 |
| H31_HUMAN | Histone H3.1 |  | FUS_HUMAN | RNA-binding protein FUS |
| H33_HUMAN | Histone H3.3 |  | FUSIP_HUMAN | FUS-interacting serine-arginine-rich protein 1 |
| H4_HUMAN | Histone H4 |  | G3P_HUMAN | Glyceraldehyde-3-phosphate dehydrogenase |
| HNRDL_HUMAN | Heterogeneous nuclear ribonucleoprotein D-like |  | H13_HUMAN | Histone H1.3 |
| HNRPC_HUMAN | Heterogeneous nuclear ribonucleoproteins C1/C2 |  | H2A1A_HUMAN | Histone H2A type 1-A |
| HNRPD_HUMAN | Heterogeneous nuclear ribonucleoprotein D0 |  | H2A1C_HUMAN | Histone H2A type 1-C |
| HNRPG_HUMAN | Processed heterogeneous nuclear ribonucleoprotein G |  | H2AY_HUMAN | Core histone macro-H2A.1 |
| HNRPK_HUMAN | Heterogeneous nuclear ribonucleoprotein K |  | H2B1B_HUMAN | Histone H2B type 1-B |
| HNRPM_HUMAN | Heterogeneous nuclear ribonucleoprotein M |  | HNRH1_HUMAN | Heterogeneous nuclear ribonucleoprotein H |
| HNRPR_HUMAN | Heterogeneous nuclear ribonucleoprotein R |  | HNRH3_HUMAN | Heterogeneous nuclear ribonucleoprotein H3 |
| HNRPU_HUMAN | Heterogeneous nuclear ribonucleoprotein U |  | HNRPL_HUMAN | Heterogeneous nuclear ribonucleoprotein L |
| HS90A_HUMAN | Heat shock protein HSP 90-alpha |  | HNRPQ_HUMAN | Heterogeneous nuclear ribonucleoprotein Q |
| MATR3_HUMAN | Matrin-3 |  | HP1B3_HUMAN | Heterochromatin protein 1-binding protein 3 |
| PARP1_HUMAN | Poly [ADP-ribose] polymerase 1 |  | HSP7C_HUMAN | Heat shock cognate 71 kDa protein |
| PCBP1_HUMAN | Poly(rC)-binding protein 1 |  | K2C1_HUMAN | Keratin, type II cytoskeletal 1 |
| PPIA_HUMAN | Peptidyl-prolyl cis-trans isomerase A |  | LMNB1_HUMAN | Lamin-B1 |
| ROA1_HUMAN | Heterogeneous nuclear ribonucleoprotein A1 |  | MIF_HUMAN | Macrophage migration inhibitory factor |
| ROA2_HUMAN | Heterogeneous nuclear ribonucleoproteins A2/B1 |  | NONO_HUMAN | Non-POU domain-containing octamer-binding protein |
| SFPQ_HUMAN | Splicing factor, proline- and glutamine-rich |  | NPM_HUMAN | Nucleophosmin |
| SFRS1_HUMAN | Splicing factor, arginine/serine-rich 1 |  | NUCL_HUMAN | Nucleolin |
| SFRS3_HUMAN | Splicing factor, arginine/serine-rich 3 |  | PGK1_HUMAN | Phosphoglycerate kinase 1 |
| SFRS7_HUMAN | Splicing factor, arginine/serine-rich 7 |  | PROF1_HUMAN | Profilin-1 |
| TBA1A_HUMAN | Tubulin alpha-1A chain |  | PSIP1_HUMAN | PC4 and SFRS1-interacting protein |
| TBB5_HUMAN | Tubulin beta chain |  | PTBP1_HUMAN | Polypyrimidine tract-binding protein 1 |
| VGF_HUMAN | Neurosecretory protein VGF |  | RALY_HUMAN | RNA-binding protein Raly |
|  |  |  | RBM39_HUMAN | RNA-binding protein 39 |
|  |  |  | RUVB1_HUMAN | RuvB-like 1 |
|  |  |  | SRP14_HUMAN | Signal recognition particle 14 kDa protein |
|  |  |  | TBB1_HUMAN | Tubulin beta-1 chain |
|  |  |  | TBB2C_HUMAN | Tubulin beta-2C chain |
|  |  |  | THOC4_HUMAN | THO complex subunit 4 |
|  |  |  | TIF1B_HUMAN | Transcription intermediary factor 1-beta |
|  |  |  | TPIS_HUMAN | Triosephosphate isomerase |
|  |  |  | U2AF2_HUMAN | Splicing factor U2AF 65 kDa subunit |
|  |  |  | UBIQ_HUMAN | Ubiquitin |
