# Supplementary material for: Amyloid domains in the cell nucleus controlled by nucleoskeletal protein lamin B1 reveal a new pathway of mercury neurotoxicity
Source: PeerJ. 2015 Feb 5;3:e754. doi: 10.7717/peerj.754 (PMC4327309; doi:10.7717/peerj.754)
Supplement: Table S3 — Mean values presented in Fig. 5C were tested for significance by one-way ANOVA and Tukey’s post-hoc test. Values indicating significance (p < 0.05) are depicted in black. [file peerj-03-754-s012.docx]

**Supplemental Table S3.** Statistical analysis of the quantification of nuclear speckle patterns.

| siRNA LB1 [pmol]  siRNA scr.  I-Hg [60 µM] | | -  -  - | 30  -  - | 60  -  - | -  +  - | -  -  + | 30  -  + | 60  -  + | -  +  + |
| --- | --- | --- | --- | --- | --- | --- | --- | --- | --- |
| **average speckle # per cell** | -  -  - |  |  |  |  |  |  |  |  |
|  | 30  -  - | 0.01502 |  |  |  |  |  |  |  |
|  | 60  -  - | 0.00309 | 0.98980 |  |  |  |  |  |  |
|  | -  +  - | 0.99400 | 0.00354 | 0.00075 |  |  |  |  |  |
|  | -  -  + | 0.94720 | 0.11816 | 0.02631 | 0.60268 |  |  |  |  |
|  | 30  -  + | 0.00574 | 0.99954 | 0.99997 | 0.00137 | 0.04826 |  |  |  |
|  | 60  -  + | 0.00266 | 0.98300 | 1.00000 | 0.00065 | 0.02271 | 1.00000 |  |  |
|  | -  +  + | 0.78001 | 0.24056 | 0.05948 | 0.36230 | 0.99981 | 0.10600 | 0.05158 |  |
| **average speckle size** | -  -  - |  |  |  |  |  |  |  |  |
|  | 30  -  - | 0.22816 |  |  |  |  |  |  |  |
|  | 60  -  - | 0.10789 | 0.99975 |  |  |  |  |  |  |
|  | -  +  - | 0.97452 | 0.72515 | 0.46345 |  |  |  |  |  |
|  | -  -  + | 0.14871 | 0.00109 | 0.00047 | 0.02577 |  |  |  |  |
|  | 30  -  + | 0.84285 | 0.92451 | 0.72421 | 0.99976 | 0.01082 |  |  |  |
|  | 60  -  + | 0.41660 | 0.99983 | 0.98481 | 0.91676 | 0.00245 | 0.99281 |  |  |
|  | -  +  + | 0.58055 | 0.00713 | 0.00298 | 0.15277 | 0.97216 | 0.06940 | 0.01623 |  |
| **average speckle shape factor** | -  -  - |  |  |  |  |  |  |  |  |
|  | 30  -  - | 0.16127 |  |  |  |  |  |  |  |
|  | 60  -  - | 0.11366 | 1.00000 |  |  |  |  |  |  |
|  | -  +  - | 0.99978 | 0.31986 | 0.23601 |  |  |  |  |  |
|  | -  -  + | 0.23073 | 0.00121 | 0.00082 | 0.11079 |  |  |  |  |
|  | 30  -  + | 0.84983 | 0.83690 | 0.73206 | 0.97584 | 0.01887 |  |  |  |
|  | 60  -  + | 0.29536 | 0.99991 | 0.99853 | 0.52286 | 0.00254 | 0.96215 |  |  |
|  | -  +  + | 0.79693 | 0.00986 | 0.00662 | 0.54508 | 0.95140 | 0.14024 | 0.02088 |  |
|  | siRNA LB1 [pmol]  siRNA scr.  I-Hg [60 µM] |  |  |  |  |  |  |  |  |
