# Supplementary material for: Amyloid domains in the cell nucleus controlled by nucleoskeletal protein lamin B1 reveal a new pathway of mercury neurotoxicity
Source: PeerJ. 2015 Feb 5;3:e754. doi: 10.7717/peerj.754 (PMC4327309; doi:10.7717/peerj.754)
Supplement: Table S4 — Mean values from Fig. 5D were tested for significance by one-way ANOVA and Tukey’s post-hoc test. Values indicating significance (p < 0.05) are depicted in bold, black lettering. [file peerj-03-754-s013.docx]

**Supplemental Table S4.** Statistical analysis of the quantification of nuclear Congo red staining patterns.

| siRNA LB1 [pmol]  siRNA scr.  I-Hg [60 µM] | | -  -  - | 30  -  - | 60  -  - | -  +  - | -  -  + | 30  -  + | 60  -  + | -  +  + |
| --- | --- | --- | --- | --- | --- | --- | --- | --- | --- |
| **CR pattern heterogeneity** | -  -  - |  |  |  |  |  |  |  |  |
|  | 30  -  - | 0.99460 |  |  |  |  |  |  |  |
|  | 60  -  - | 0.99048 | 0.78083 |  |  |  |  |  |  |
|  | -  +  - | 1.00000 | 0.99753 | 0.98285 |  |  |  |  |  |
|  | -  -  + | 1.10*10^-7^ | 1.27*10^-7^ | 1.46*10^-7^ | 1.12*10^-7^ |  |  |  |  |
|  | 30  -  + | 1.48*10^-7^ | 1.46*10^-7^ | 1.16*10^-7^ | 1.53*10^-7^ | 0.93051 |  |  |  |
|  | 60  -  + | 3.65*10^-6^ | 0.00001 | 1.06*10^-6^ | 4.18*10^-6^ | 0.01987 | 0.16846 |  |  |
|  | -  +  + | 1.14*10^-7^ | 1.40*10^-7^ | 1.44*10^-7^ | 1.16*10^-7^ | 0.99999 | 0.98186 | 0.03321 |  |
| **CR pattern intensity** | -  -  - |  |  |  |  |  |  |  |  |
|  | 30  -  - | 0.99686 |  |  |  |  |  |  |  |
|  | 60  -  - | 1.00000 | 0.99961 |  |  |  |  |  |  |
|  | -  +  - | 0.99857 | 1.00000 | 0.99988 |  |  |  |  |  |
|  | -  -  + | 5.31*10^-6^ | 0.00002 | 7.10*10^-6^ | 0.00001 |  |  |  |  |
|  | 30  -  + | 8.87*10^-6^ | 0.00003 | 0.00001 | 0.00002 | 0.99997 |  |  |  |
|  | 60  -  + | 0.01468 | 0.05260 | 0.02105 | 0.04839 | 0.00778 | 0.01468 |  |  |
|  | -  +  + | 0.00001 | 0.00003 | 0.00001 | 0.00003 | 0.99979 | 1.00000 | 0.01826 |  |
| **nucleus area with aggregates** | -  -  - |  |  |  |  |  |  |  |  |
|  | 30  -  - | 1.00000 |  |  |  |  |  |  |  |
|  | 60  -  - | 1.00000 | 1.00000 |  |  |  |  |  |  |
|  | -  +  - | 1.00000 | 1.00000 | 1.00000 |  |  |  |  |  |
|  | -  -  + | 5.00*10^-6^ | 6.55*10^-6^ | 5.37*10^-6^ | 5.80*10^-6^ |  |  |  |  |
|  | 30  -  + | 0.00001 | 0.00002 | 0.00001 | 0.00001 | 0.99862 |  |  |  |
|  | 60  -  + | 0.18146 | 0.23857 | 0.19538 | 0.21119 | 0.00053 | 0.00159 |  |  |
|  | -  +  + | 0.00001 | 0.00001 | 0.00001 | 0.00001 | 0.99962 | 1.00000 | 0.00129 |  |
|  | siRNA LB1 [pmol]  siRNA scr.  I-Hg [60 µM] |  |  |  |  |  |  |  |  |
