# Supplementary material for: Amyloid domains in the cell nucleus controlled by nucleoskeletal protein lamin B1 reveal a new pathway of mercury neurotoxicity
Source: PeerJ. 2015 Feb 5;3:e754. doi: 10.7717/peerj.754 (PMC4327309; doi:10.7717/peerj.754)
Supplement: Data S5 [file peerj-03-754-s018.pdf]

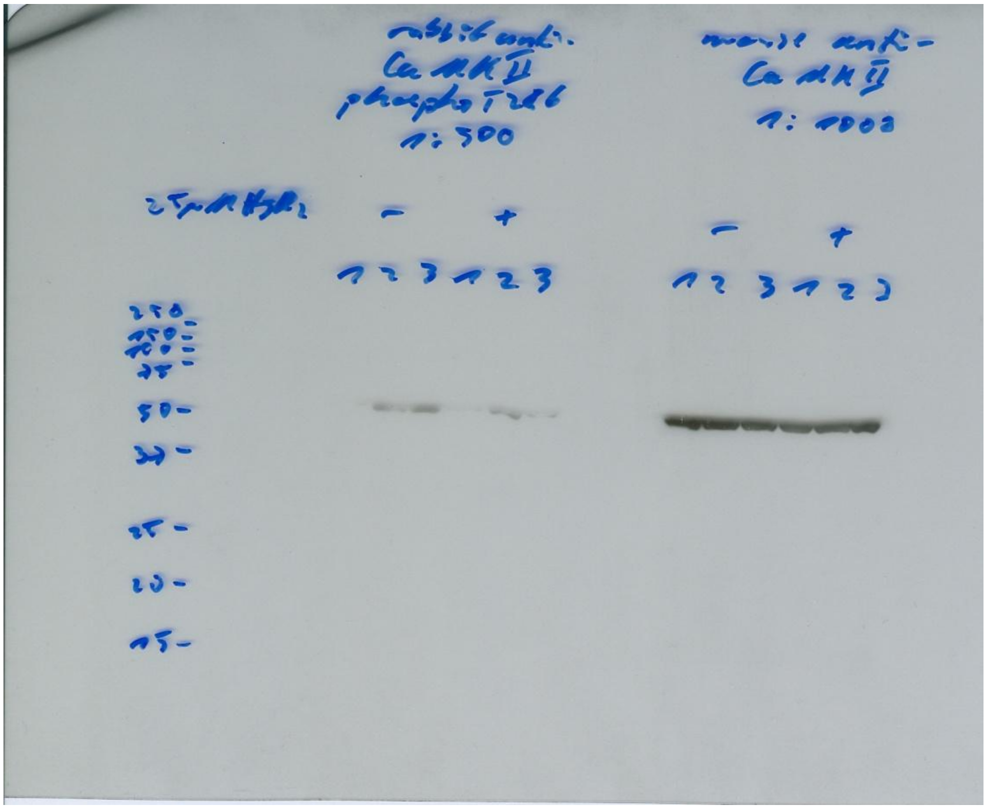
rabbit anti-
CaMKII
phospho T286
1:500
mouse anti-
CaMKII
1:1000
25 µM H2O2
- +
- +
1 2 3 1 2 3
1 2 3 1 2 3
250
150
100
75
50
37
25
20
15

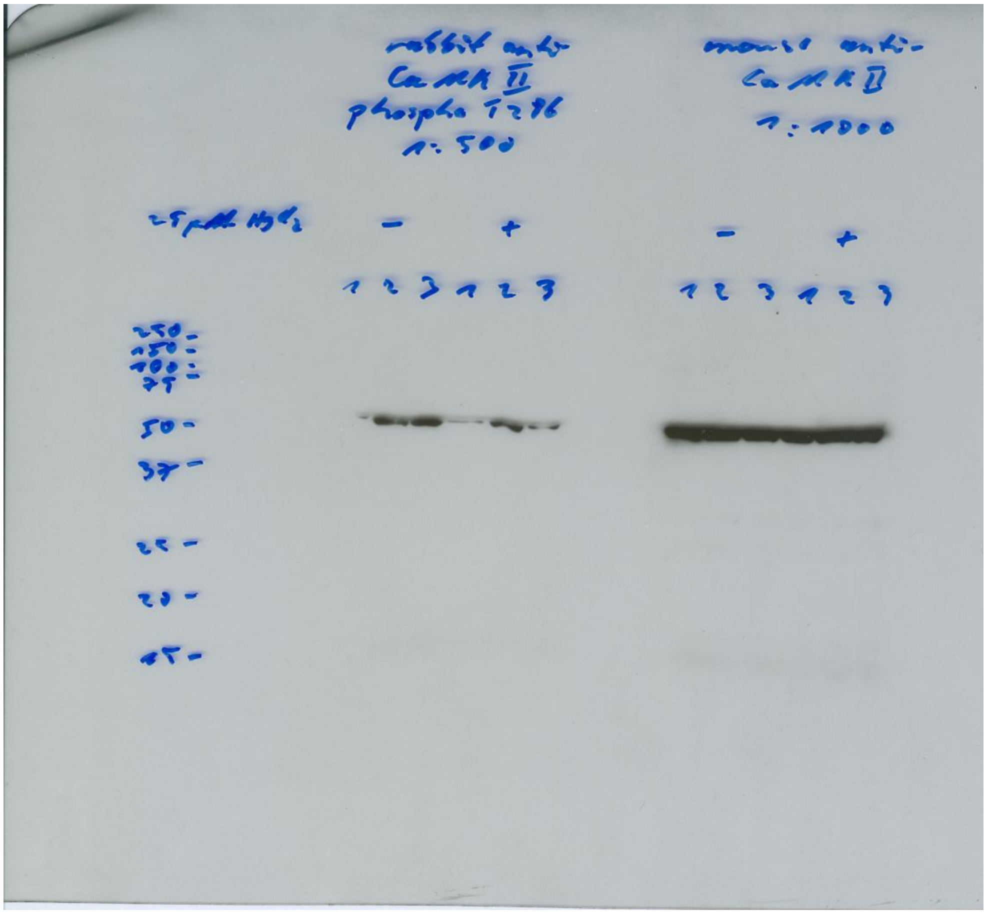
rabbit anti-
CaMKII
phospho T286
1:500
mouse anti-
CaMKII
1:1000
25 µM H2O2
- +
- +
1 2 3 1 2 3
1 2 3 1 2 3
250
150
100
75
50
37
25
20
15

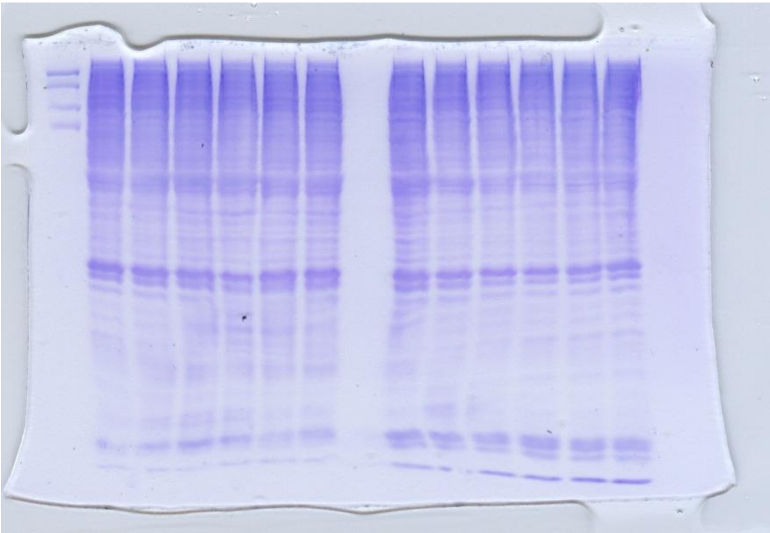

Raw data 1. Original Western blot Figure 1. Detection of CaMKII phospho T286 and total CaMKII in unstimulated and stimulated SH-SY5Y cells (untreated or pretreated with I-Hg).

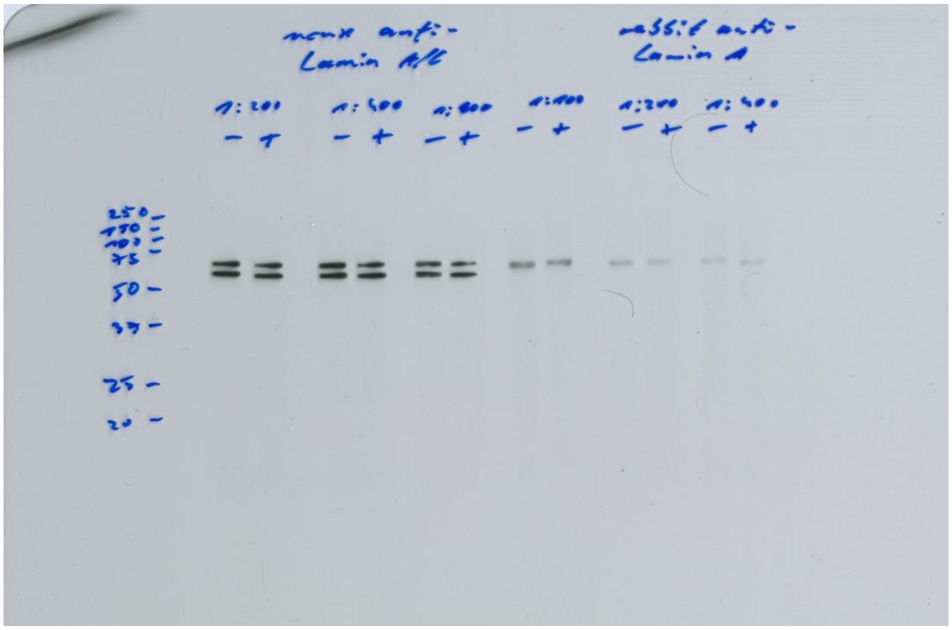

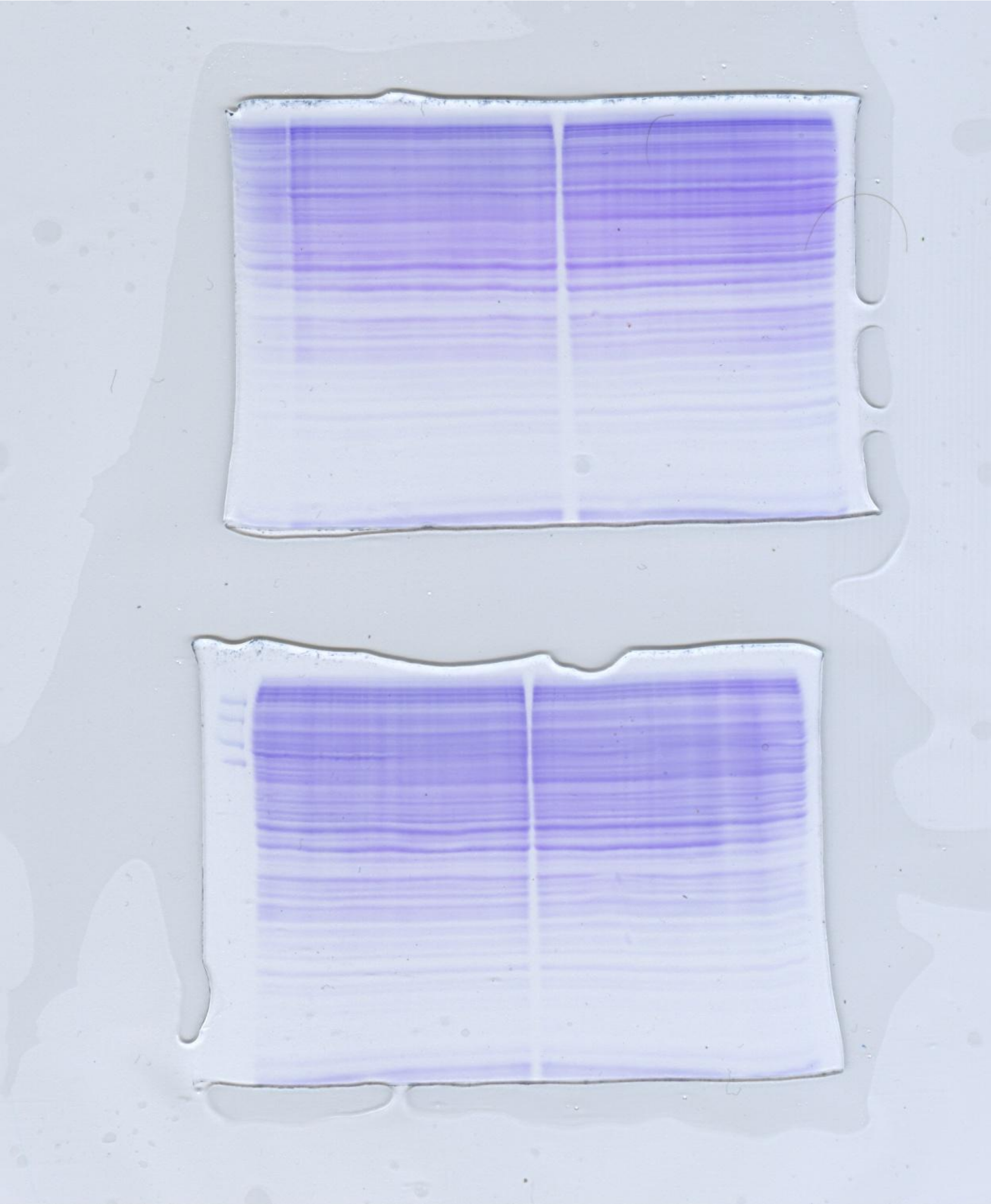

Raw data 2. Original Western blot Figure 4. Detection of Lamin A/C in untreated or I-Hg-treated HEp-2 cells.

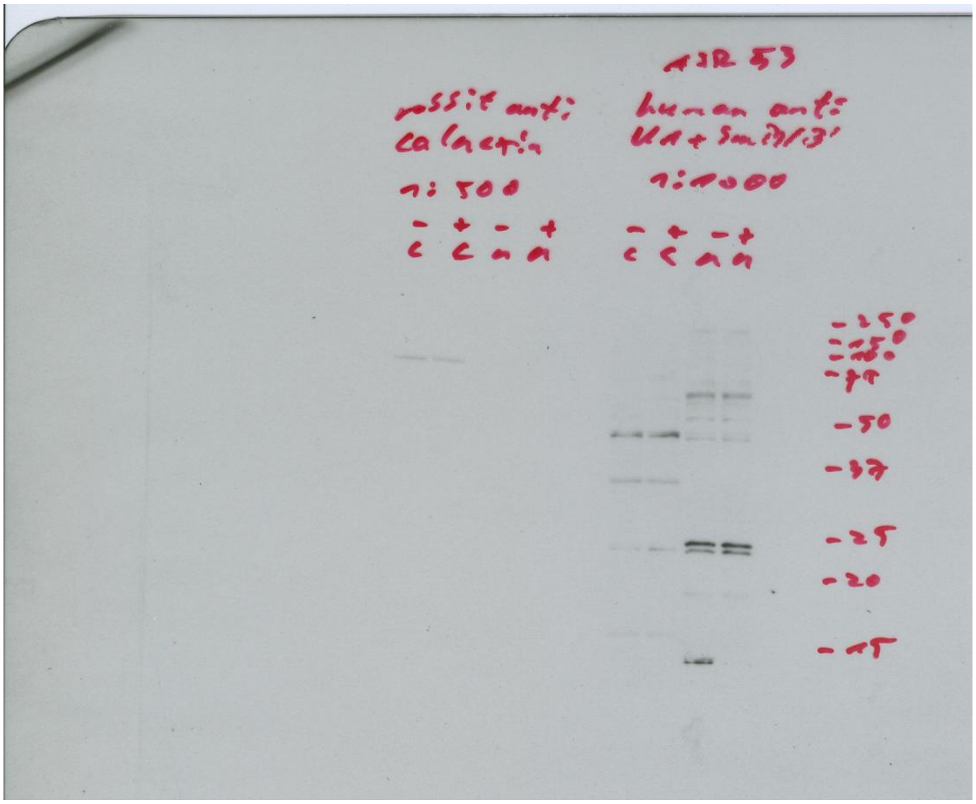

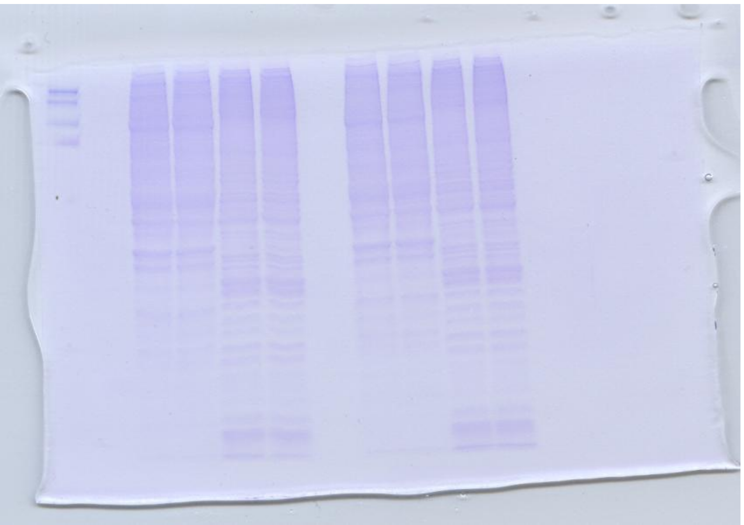

Raw data 3. Original Western blot Supplemental Figure S2. HEp-2 fractionation control.

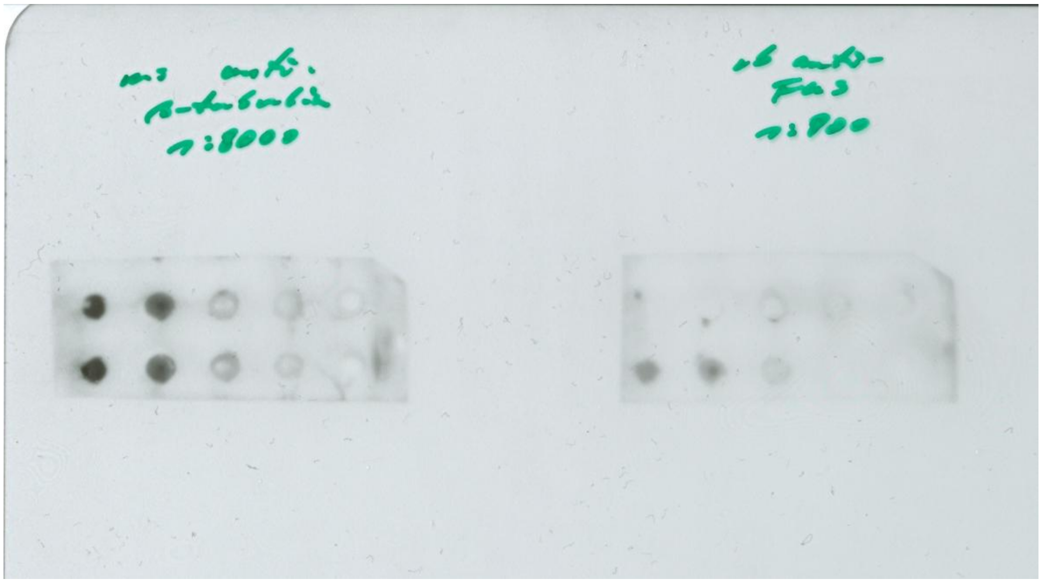
ms anti-
β-tubulin
1:8000
rb anti-
FUS
1:900

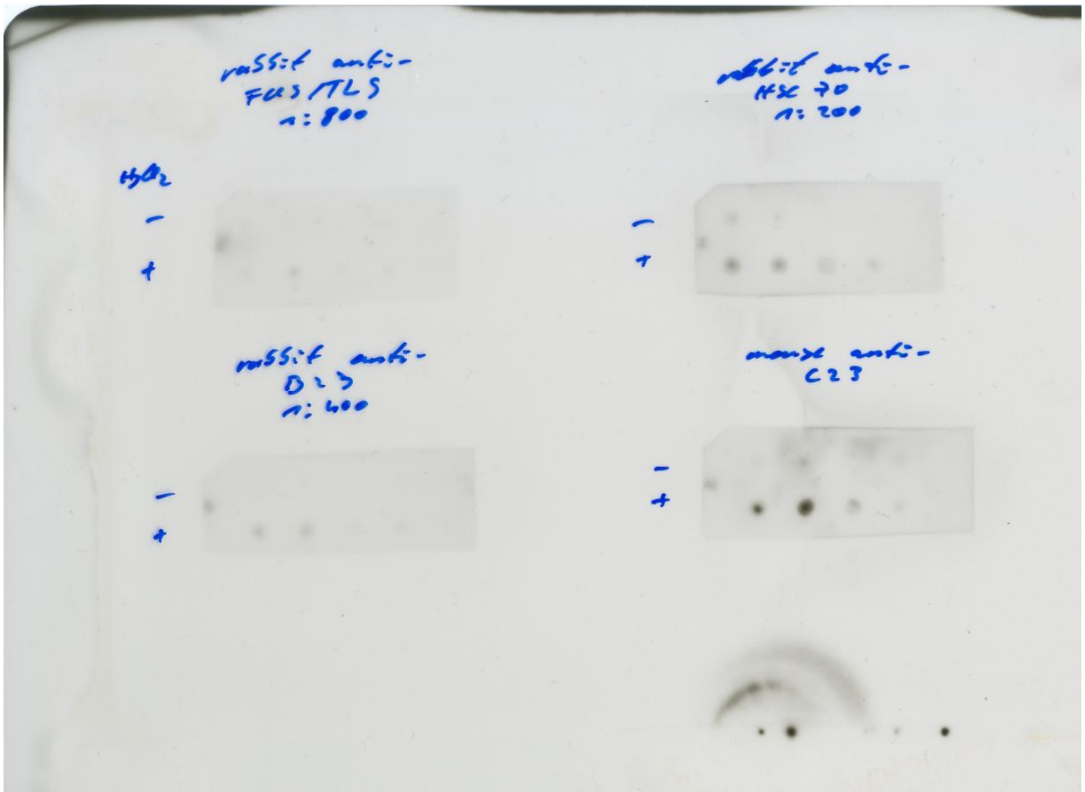
rabbit anti-
FUS/TLS
1:800
rabbit anti-
HSC 70
1:200
H_2O_2
–
+
–
+
rabbit anti-
B23
1:400
mouse anti-
C23
–
+
–
+

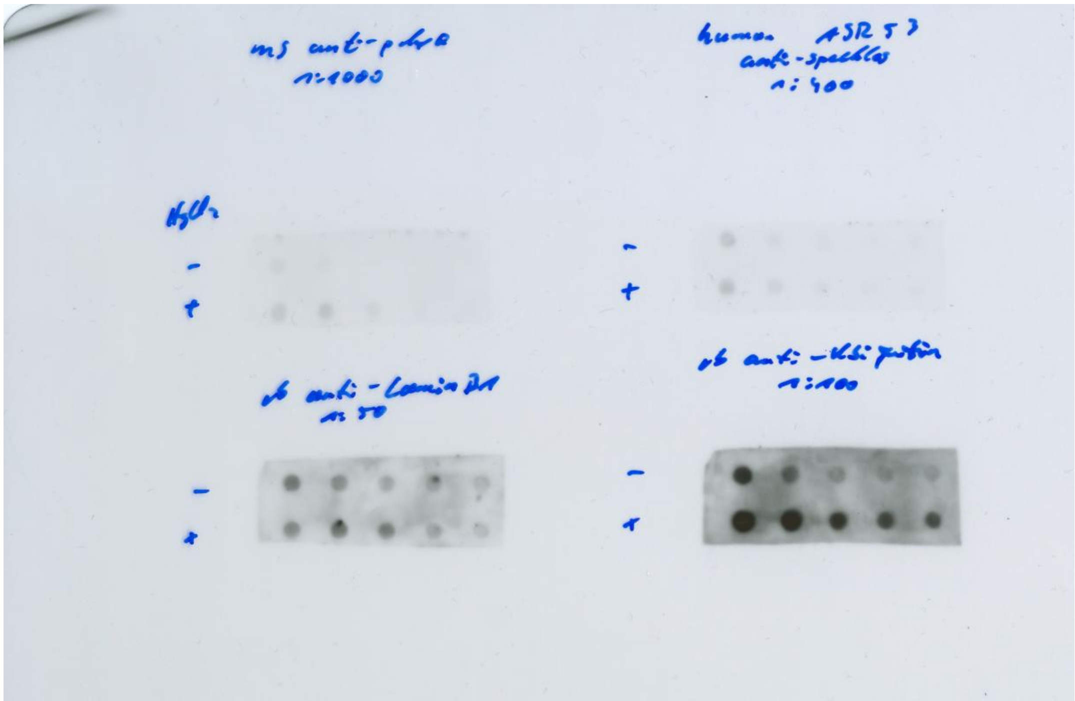

Raw data 4. Original filter retardation assay Supplemental Figure S4. Untreated or I-Hg-treated SH-SY5Y.

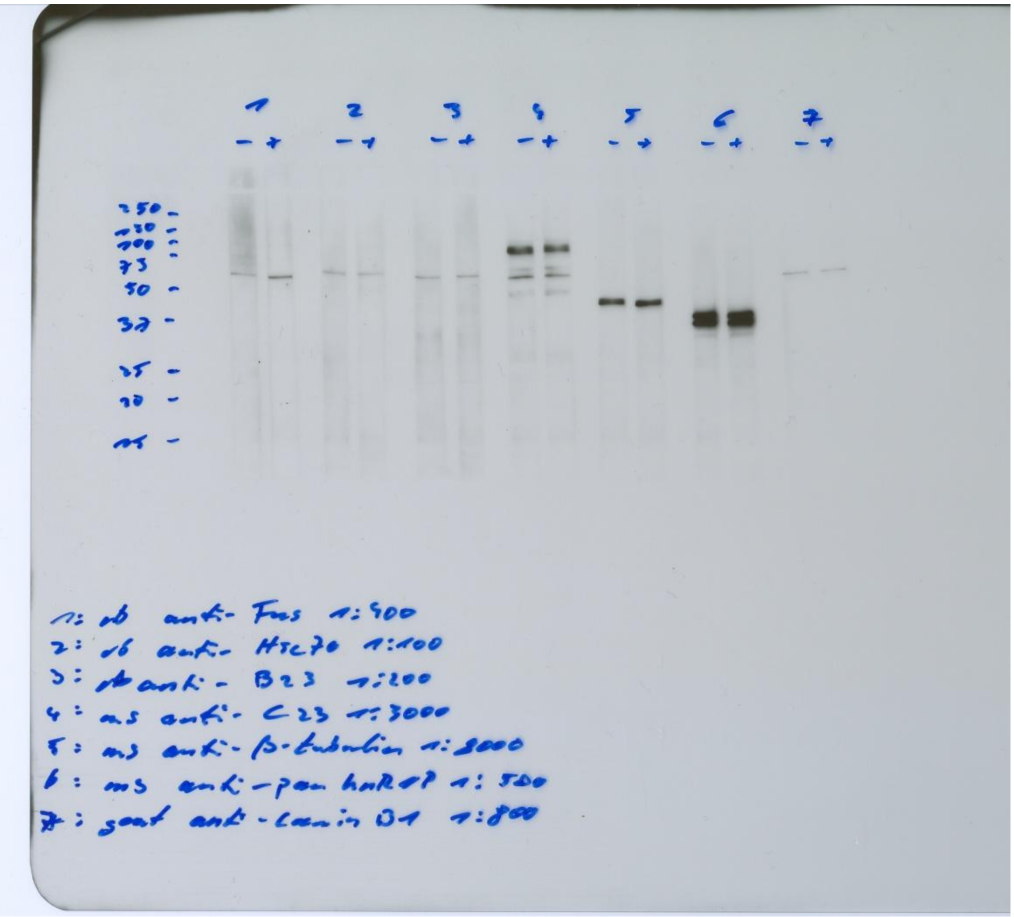

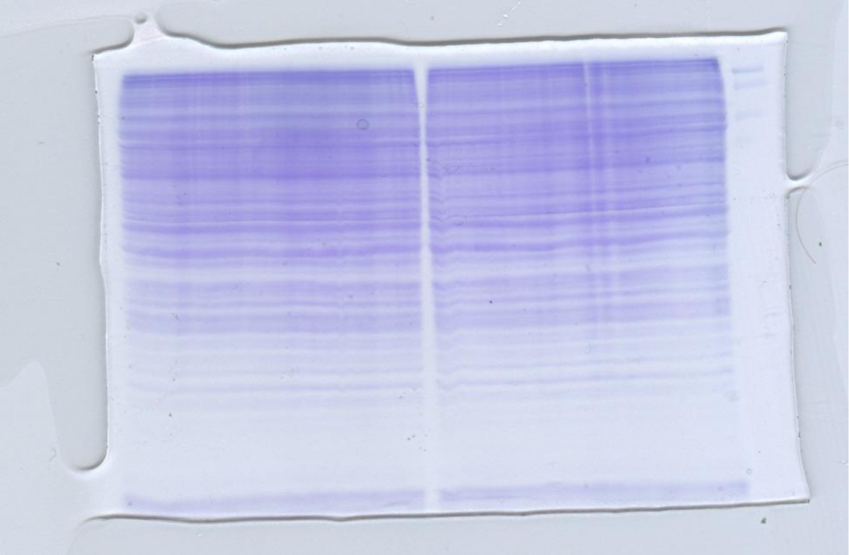

Raw data 5. Original Western blot Supplemental Figure S4. Untreated or I-Hg-treated SH-SY5Y.

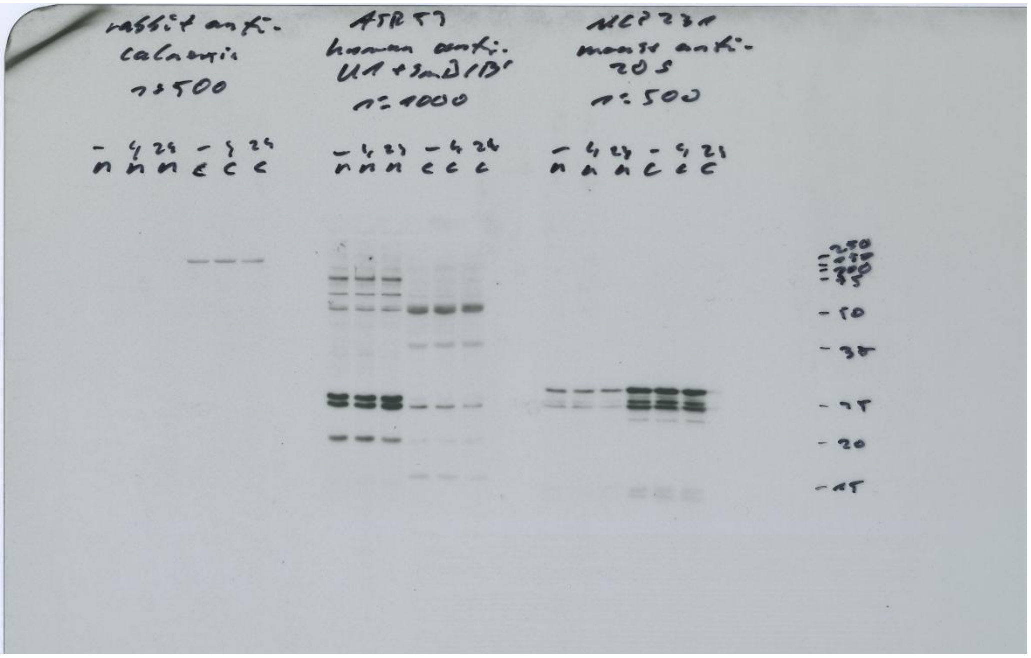
rabbit anti-
calnexin
1:500
ASR 57
human anti-
U1 + SmB/B'
1:1000
MCP 231
mouse anti-
20S
1:500
- 4 24 - 4 24
n n n c c c
- 50
- 37
- 25
- 20
- 15

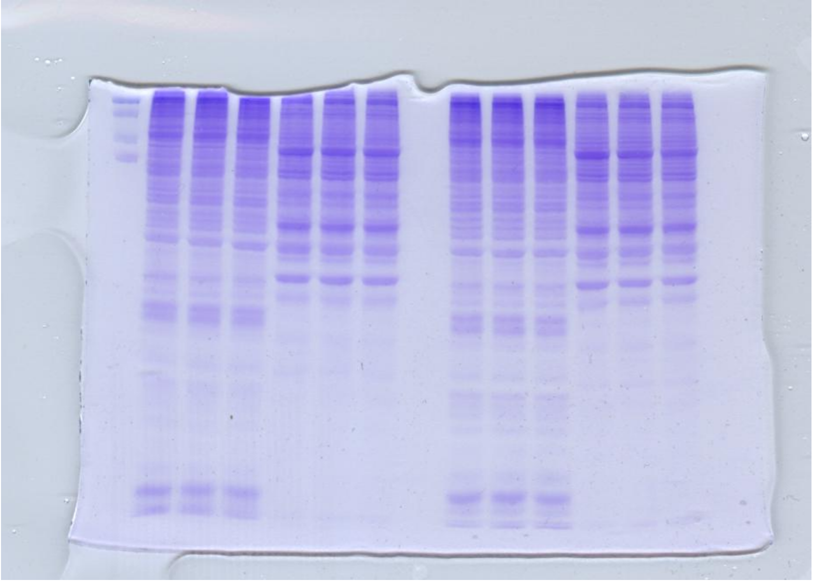

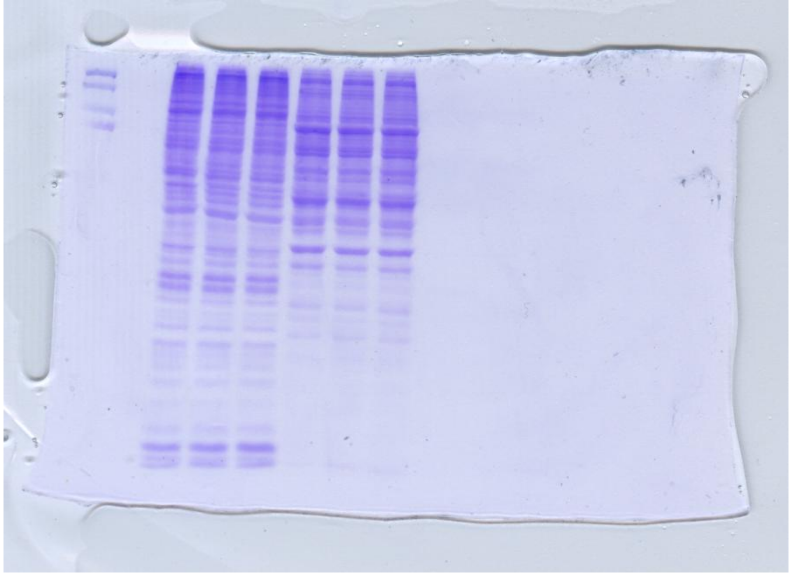

Raw data 6. Original Western blot Supplemental Figure S6. Untreated or I-Hg-treated (4h, 24h) HEp-2.

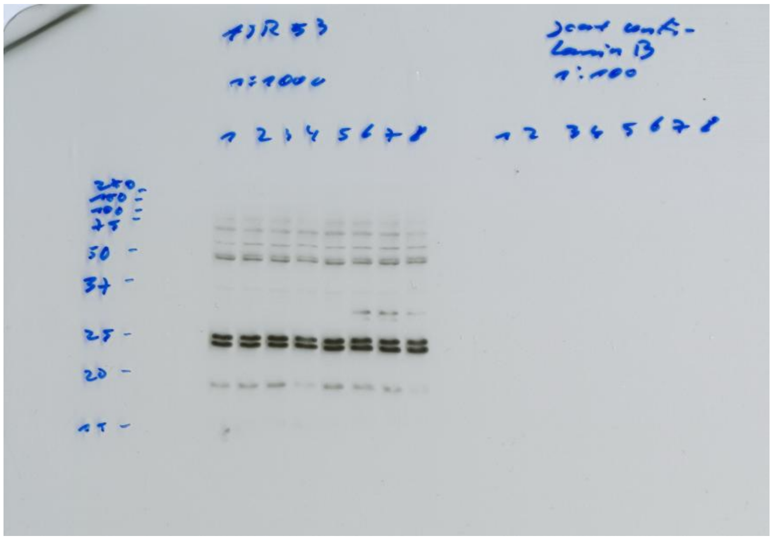

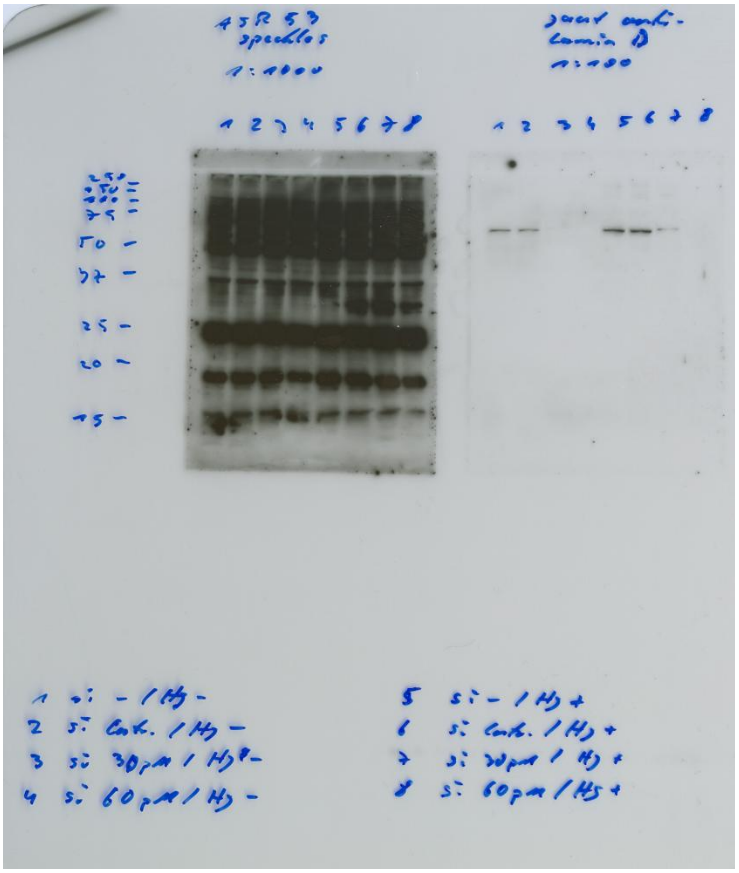

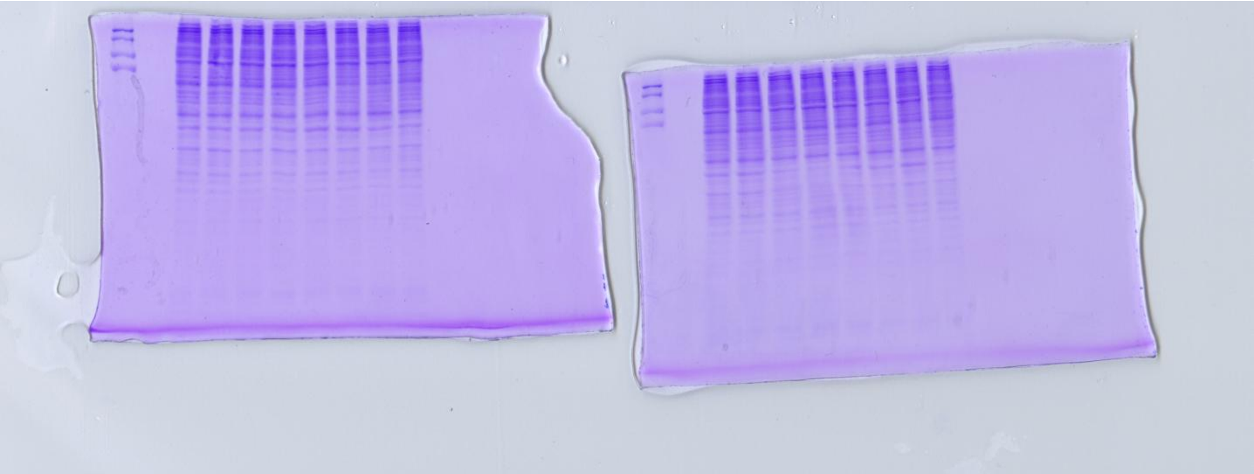

Raw data 7. Original Western blot Supplemental Figure S8. Untreated or siRNA-pretreated HEp-2 (untreated or I-Hg-treated).
